# Marine-derived extracts of *Peyssonnelia caulifera Okamura* and *Meristotheca papulosa* demonstrate differential efficacy in modulating obesity-related metabolic skewing revealed by integrative analysis of extract metabolomics and microbiome profiles

**DOI:** 10.3389/fnut.2026.1749413

**Published:** 2026-04-09

**Authors:** Fang Feng, Jihye Choi, Dohyun Ahn, Thi My Tien Truong, Kyung June Yim, Hyun-Jin Kim, Man-Young Jung, Hae-Won Lee, Dong-Shin Kim, Inhae Kang

**Affiliations:** 1 Department of Food Science and Nutrition, Jeju National University, Jeju, Republic of Korea; 2 Department of Food Bioengineering, Jeju National University, Jeju, Republic of Korea; 3 Department of Food Science and Technology, Gyeongsang National University, Jinju, Republic of Korea; 4 Division of Food Science and Technology, Institute of Agriculture and Life Science, Gyeongsang National University, Jinju, Republic of Korea; 5 Interdisciplinary Graduate Program in Advanced Convergence Technology and Science, Jeju National University, Jeju, Republic of Korea

**Keywords:** gut microbiota, *Meristotheca papulosa*, metabolomics, metainflammation, obesity, *Peyssonnelia caulifera Okamura*

## Abstract

This study investigated the anti-obesity efficacy of *Peyssonnelia caulifera Okamura* extract (PCE) and *Meristotheca papulosa* extract (MPE) in a high-fat diet (HFD)-induced obese mouse model. Both extracts improved hyperinsulinemia, adipocyte hypertrophy, and adipose/hepatic inflammation. PCE significantly reduced fasting glucose and hepatic triglyceride levels, while MPE effectively normalized colonic histopathology. Both extracts restored tight junction protein expression and mitigated gut barrier disruption. At the phylum level, both supplementations decreased *Bacteroidota* and increased *Verrucomicrobiota*; At the genus level, MPE significantly enriched *Lachnospiraceae NK4A136*, *Dubosiella*, *Faecalibaculum*, and *Ruminococcaceae NK4A214*, while PCE showed modest, non-significant increase. PCE more potently suppressed LPS-induced cytokines expression and adipogenesis than MPE *in vitro*. UPLC-QTOF-MS revealed distinct metabolite fingerprints for each extract, and correlation analysis linked key metabolites (e.g., carnitine, valyl isoleucine) to inflammatory and metabolic indices. These findings identify PCE and MPE confer metabolic benefits in HFD-induced obesity through coordinated effects on gut, hepatic, and adipose tissue responses, with PCE showing superior efficacy.

## 1 Introduction

Obesity is a substantial global health and economic challenge, affecting over 650 million adults and 340 million children worldwide, and costing an estimated $2 trillion annually (1). Obesity is intricately linked to a spectrum of metabolic complications, substantially increasing the risk and severity of comorbid diseases, such as type 2 diabetes, hypertension, cardiovascular diseases, metabolic dysfunction-associated fatty liver disease (MAFLD), and certain cancers (2, 3). Excess adipose tissue promotes chronic low-grade inflammation and insulin resistance. The synergistic effects of obesity and metabolic diseases underscore the urgency of addressing obesity as an isolated condition and a primary driver of metabolic dysregulation. The pharmaceutical market for obesity management is rapidly expanding, offering drugs that target appetite suppression, energy expenditure, and fat absorption [e.g., orlistat, phentermine, and glucagon-like peptide-1 (GLP-1) receptor agonists]. However, many of these treatments are associated with side effects, including gastrointestinal disturbances, cardiovascular risks, and neuropsychiatric symptoms, which limit their widespread use (4, 5). Therefore, bioactive compounds derived from edible plants and marine resources are promising alternatives to mitigating obesity and its associated complications (6, 7).

The gut plays a pivotal role in maintaining overall health, and gut integrity and microbiome balance are crucial in obesity (8). The intestinal barrier, consisting of epithelial cells and tight junction proteins, acts as a physical and immunological shield, sequestering harmful microbes and endotoxins from the systemic circulation (9). In obesity, this barrier is compromised, resulting in increased gut permeability or a “leaky gut,” which facilitates the translocation of microbial components, such as lipopolysaccharides (LPS), into the bloodstream. This triggers low-grade chronic inflammation, a major driver of metabolic perturbations associated with obesity (10). Dysbiosis of the gut microbiome is characterized by diminished microbial diversity and a surge in proinflammatory species. This imbalance exacerbates intestinal inflammation, disrupts energy homeostasis, and skews lipid metabolism, thereby contributing to inflammation (11). The interplay between gut barrier dysfunction and microbiome imbalance is critical in obesity-related pathophysiology. Thus, identifying the bioactive molecules that modulate gut integrity and microbiome composition is important for targeting obesity-induced inflammation and associated metabolic disorders.

Seaweeds, rich sources of bioactive components, have garnered considerable attention owing to their health-promoting properties. Their diverse metabolite profiles, including those of polysaccharides, polyphenols, and carotenoids, exhibit a myriad of biological activities, such as anti-inflammatory, anti-adipogenic, and antioxidant effects (12–14). These bioactivities are attributed to their ability to modulate various metabolic pathways and cellular signaling networks, making seaweed-derived extracts promising candidates for therapeutic and nutritional applications. The bioactive components of seaweed extracts, such as polyunsaturated fatty acids, fucoxanthin (15), astaxanthin (16), and phenolic compounds, such as dieckol (17), inhibit lipid accumulation during adipogenesis, attenuate inflammatory responses, and improve metabolic health through their unique metabolite compositions. Among marine sources, red algae species, such as *Peyssonnelia caulifera* (PC) and *Meristotheca papulosa* (MP), which typically thrive in warm regions, such as Jeju, South Korea and southern Japan, remain underexplored for their health benefits. Ho et al. demonstrated that PC extract (PCE) enhances the efficacy of an inactivated H1N1 influenza vaccine in a murine model by stimulating balanced Th1/Th2 responses, memory T and B cells, and dendritic cell activation (18). Moreover, the immunostimulatory effects of both PCE and MP extracts (MPE) promote the activity of antigen-presenting cells (19). However, their potential roles in the mitigation of obesity and meta-inflammation remain largely unknown.

In this study, we investigated the effects of PCE and MPE on obesity-related metabolic complications using a high-fat diet (HFD) model in C57BL/6 mice. Key outcomes, including visceral adiposity, hepatic lipid accumulation, inflammatory gene expression, intestinal barrier integrity, and microbiome composition, were evaluated to elucidate the therapeutic potential within the gut-liver-adipose axis. Additionally, LPS-induced inflammatory macrophages and adipocytes were used *in vitro* to delineate the distinct roles of PCE and MPE. Metabolomic profiling was used to identify unique bioactive compounds and their differential metabolic pathways. This study advances our understanding of marine-derived therapeutics for metabolic disorders, and provides a foundation for the development of targeted natural interventions for obesity and its associated complications.

## 2 Materials and methods

### 2.1 Experimental materials and sample preparation

PCE and MPE were obtained using a 50% ethanol extraction method and provided by the Marine BioBank of the Marine Biodiversity Institute of Korea (Seocheon, Korea [http://www.mabik.re.kr/biobank]). Dried seaweed materials were extracted with 50% ethanol and the resulting solutions were filtered, concentrated, and lyophilized to yield dry extracts. These were designated as the PCE and MPE, respectively. The extracts were dissolved in dimethyl sulfoxide (DMSO; Sigma-Aldrich, St. Louis, MO, USA) at a stock concentration of 80 mg/mL, aliquoted, and stored at −20 °C. Fresh aliquots were used for each experiment. All reagents were purchased from Sigma-Aldrich unless otherwise stated.

### 2.2 Measurement of total polyphenol and flavonoid contents and radical scavenging activities

Total polyphenol content (TPC) was determined using the Folin–Ciocalteu colorimetric method as previously described (20). Folin–Ciocalteu reagent was diluted 1:10 (v/v) with distilled water. A 4% (w/v) Na_2_CO_3_ solution was prepared (400 mg anhydrous Na_2_CO_3_ in 10 mL water). Gallic acid standards (0, 6.25, 12.5, 25, 50, and 100 μg/mL) were prepared in water. In 96-well plates, 10 μL of standard or appropriately diluted sample was mixed with 75 μL of diluted Folin reagent and incubated for 5 min at room temperature. Then, 75 μL of 4% Na_2_CO_3_ was added, and the mixture was incubated for 1 h in the dark. Absorbance was read at 725 nm. To correct pigment/turbidity, a sample blank (sample processed identically with water replacing Folin reagent) was included when necessary. TPC was calculated from the gallic acid standard curve and expressed as mg gallic acid equivalents per g dry extract (mg GAE/g). All measurements were performed in triplicate.

Total flavonoid content (TFC) was measured using the NaNO_2_–AlCl_3_–NaOH colorimetric method (21). Solutions were prepared as follows: 5% NaNO_2_ (50 mg/mL), 10% AlCl_3_ (100 mg/mL), and 1 M NaOH (400 mg/10 mL). Catechin standards (0, 0.0125, 0.025, 0.05, 0.1, and 0.2 mg/mL) were prepared in methanol. In 96-well plates, 100 μL of standard or sample was mixed with 50 μL water and 15 μL of 5% NaNO_2_ and incubated for 6 min at room temperature. Then, 30 μL of 10% AlCl_3_ was added and incubated for 5 min, followed by addition of 100 μL of 1 M NaOH. Absorbance was measured at 510 nm. For pigmented samples, a sample blank (prepared identically but omitting AlCl_3_) was included when necessary. TFC was calculated from the catechin standard curve and expressed as mg catechin equivalents per g dry extract (mg CE/g). All measurements were performed in triplicate.

NaCl-equivalent salt content of seaweed extracts was measured using a digital salinity meter (Scionix SSM-500, China; Range 0–5% w/v), which estimates salinity based on electrical conductivity. The instrument was calibrated with NaCl standards (0, 1, 2, and 4% w/v), each measured in triplicate, and linearity was verified by regression of meter readings against nominal NaCl concentrations. Extract powders (PCE and MPE) were dissolved in deionized water to 0.5 mg/mL, vortex-mixed to homogeneity, and equilibrated at room temperature (~25 °C) for 5–10 min. Salinity was recorded after the reading stabilized with the probe immersed in the solution while avoiding contact with the container walls. All measurements were performed in triplicate.

The 2,2-Diphenyl-1-picryl-hydrazyl-hydrate (DPPH) radical scavenging activity was measured using a microplate-based method (22). A 10 mM DPPH stock was prepared in absolute ethanol and diluted to 0.2 mM immediately before use (protected from light). In 96-well plates, 25 μL of seaweed extract (or L(+)-ascorbic acid, AA) was mixed with 175 μL of 0.2 mM DPPH. Sample blanks contained 25 μL sample + 175 μL ethanol, and controls contained 25 μL ethanol + 175 μL DPPH. After 30 min incubation at room temperature in the dark, absorbance was read at 517 nm. All measurements were performed in triplicate. Scavenging activity was calculated as:


$$\%\text{scavenging}=1-(\frac{A_{\text{sample}}-A_{\text{blank}}}{A_{\text{control}}})\times 100$$

The 2,2′-azino-bis(3-ethylbenzothiazoline-6-sulfonic acid) radical cation (ABTS) scavenging activity was assessed as described (23). ABTS was generated by mixing 7.4 mM ABTS (10 mg in 2.46 mL water) with 2.6 mM potassium persulfate (35.14 mg in 50 mL water) at 1:1 (v/v) and incubating 12–24 h at room temperature in the dark. The radical solution was diluted with water to an absorbance of 1.4–1.5 at 735 nm. In 96-well plates, 25 μL of seaweed extracts (or AA) was combined with 175 μL ABTS working solution. Color controls contained 25 μL sample (or AA) + 175 μL water, and controls contained 25 μL water + 175 μL ABTS After 30 min incubation at room temperature in the dark, absorbance was measured at 735 nm. Scavenging activity was calculated using the same equation above. All measurements were performed in triplicate.

### 2.3 Animals diets and treatment

All protocols and procedures were approved by the Institutional Animal Care and Use Committee of Jeju National University (Approval ID # 2022–0057). The 6-week-old male C57BL/6 J mice were purchased from the ORIENT BIO Animal Center (Seongnam-si, Korea), housed at Jeju National University under controlled environmental conditions with a 12 h light/12 h dark cycle, and allowed to consume water and a standard chow diet *ad libitum*. The mice were divided into four groups: low-fat diet (LF, 11% calories from fat, *n* = 10), high-fat (HF; 61% calories from fat, *n* = 9), HF with PCE (HF + PCE, *n* = 10), or HF with MPE (*n* = 8). The animals were provided *ad libitum* access to these diets, and PCE and MPE were orally administered at a dose of 10 mg/kg body weight (BW) once every 24 h for five consecutive days during 8 weeks of experimental period. The AIN-93G diet was used as the LF control diet, and the HFD was modified from the AIN-93G and a typical HFD (61% calories from fat; Supplementary Table S1). BW was measured weekly until week 10. Food and drink intake was evaluated three times a week from the fifth week to the end of the experiment.

### 2.4 Glucose and insulin tolerance test

For the glucose tolerance test (GTT) and insulin tolerance test (ITT), mice were fasted (12 h) before intraperitoneal injection of 10% D-glucose solution (0.5 g/kg BW) or insulin (0.75 U/kg BW), respectively. Blood glucose levels were measured before injection and at 20, 40, 60, and 120 min after injection using a Contour Plus Glucometer (Bayer, NY, USA). The homeostatic model assessment for insulin resistance (HOMA-IR) was calculated as:


$$\text{HOMA}-\mathrm{IR}=[\begin{array}{l} \text{fasting plasma glucose}\;(\mathrm{mg}/\mathrm{dl})\\ \times \text{fasting plasma insulin}\;(\mathrm{mIU}/L) \end{array}]/405\;(\text{mmol}/L)$$

### 2.5 Serum biochemistry

After completion of the experiment, the animals were fasted for 12 h and euthanized via carbon dioxide narcosis. Blood was collected via cardiac puncture and serum samples were aliquoted. Serum total cholesterol (TC, mg/dL) and triglyceride (TG, mg/dL) levels were analyzed using an enzyme assay kit (Asan Pharmaceutical Co., Seoul, Korea) according to the manufacturer’s protocol, with absorbance measured at wavelengths of 500 and 550 nm, respectively. Serum aspartate aminotransferase (AST, IU/L) and alanine aminotransferase (ALT, IU/L) levels were measured at an absorbance wavelength of 505 nm using a kit (Asan Pharmaceutical Co.). Insulin levels in mouse serum were measured using an ultrasensitive mouse insulin ELISA kit according to the manufacturer’s protocol (Crystal Chem, Elk Grove Village, IL, USA).

### 2.6 Histopathological analysis

Epididymal adipose tissue, liver, and colon samples were collected during necropsy and fixed in 10% neutral-buffered formalin for 24–48 h. Following standard procedures, tissues were paraffin-embedded, sectioned at 3–5 μm, and stained with hematoxylin and eosin (H&E). Histological images were captured using a digital inverted fluorescence microscope (EVOS FL, Invitrogen, Carlsbad, CA, USA) at 20× and 40× magnifications. Adipocyte size and area were quantified using ImageJ software (National Institutes of Health, Bethesda, MD, USA). For each sample, at least five randomly selected fields were processed using binarization and watershed segmentation, and the average adipocyte area (μm^2^) was calculated. Liver sections were evaluated semi-quantitatively for steatosis, lobular inflammation, and hepatocellular ballooning based on scoring criteria (24). Colon sections were assessed for immune cell infiltration and epithelial damage according to previously described criteria. Infiltration was graded on a scale of 1–4, based on the depth and distribution of immune cells across the mucosal layers. Epithelial damage was scored from 0 to 4 according to epithelial elongation relative to the nuclear diameter (25).

### 2.7 Hepatic lipid accumulation

Total hepatic lipids were extracted from 200 mg of the liver tissue using methanol/chloroform (1:2 volume [v/v]). The samples were incubated at room temperature, and then at 60 °C for 3 h. The following day, the extracts were filtered through a Whatman filter paper. The samples were washed several times with chloroform and mixture was dried at room temperature. The obtained samples were resuspended in deionized water. To measure the TG and TC content in the liver, enzyme assay kits for TG and TC (Asan Pharmaceutical Co.) were used with absorbance at wavelengths of 550 and 500 nm, respectively.

### 2.8 Cell cultures

RAW264.7 murine macrophage-like cells were purchased from Korea Cell Line Bank (KCLB, Seoul, Korea) and cultured as previously describe (26). RAW264.7 murine macrophage cells were seeded at a density of 1 × 10^5^ cells/well in 96-well plates and cultured in Dulbecco’s Modified Eagle Medium (DMEM) supplemented with 10% fetal bovine serum (FBS) and 1% penicillin–streptomycin at 37 °C in a humidified atmosphere containing 5% CO_2_. To evaluate cytotoxicity, cells were treated with various concentrations (5–40 μg/mL) of PCE or MPE for 24 h, and cell viability was assessed using the MTT assay. For nitric oxide (NO) production analysis, cells were pretreated with PCE or MPE (5–20 μg/mL) for 1 h, followed by stimulation with lipopolysaccharide (LPS, 1 μg/mL) for 24 h. The NO concentration in the culture supernatant was measured using Griess reagent, and absorbance was recorded at 540 nm using a microplate reader.

The 3 T3-L1 cells were purchased from American Type Culture Collection (ATCC, VA, USA) and grown as described previously (26). For differentiation of 3 T3-L1 cells, 3 T3-L1 preadipocytes were maintained in DMEM supplemented with 10% bovine calf serum until confluence. Adipogenic differentiation was induced by treating the cells with a differentiation cocktail (MDI) containing 0.5 mM 3-isobutyl-1-methylxanthine (IBMX), 1 μM dexamethasone, and 2 nM insulin for 2 days. This was followed by treatment with insulin-only medium for an additional 6 days. PCE or MPE (5, 10, or 20 μg/mL) was added throughout the entire differentiation period. On day 8, intracellular lipid accumulation was evaluated using Oil Red O (ORO) staining. Cells were fixed with 10% formalin, stained with 0.35% ORO solution for 10 min at room temperature, and imaged under a light microscope. For quantitative analysis, the dye was eluted with isopropanol, and absorbance was measured at 500 nm.

### 2.9 Analysis of mRNA using real-time-polymerase chain reaction (PCR)

At the end of the experiment, 0.2 g of fat, liver, and colon tissue was rapidly stored in a freezer, and cells were subjected to RNA extraction using TRIzol reagent (Invitrogen). cDNA was synthesized using a high-capacity cDNA reverse transcription kit (Applied Biosystems, Foster City, CA, USA) after measuring the concentration of extracted RNA using a NanoDrop Nano-200 Micro-Spectrophotometer (Hangzhou City, China). Gene expression was determined using real-time PCR (CFX96 Real-Time PCR Detection System, Bio-Rad, Hercules, CA, USA). Relative gene expression was normalized to hypoxanthine-guanine phosphoribosyltransferase (HPRT) and/or ribosomal protein lateral stalk subunit P0 (RPLP0, 36B4) (Cosmo Genetech; Supplementary Table S2).

### 2.10 DNA isolation and 16S rRNA amplicon sequencing

Before sacrifice, fecal samples were collected in sterile Eppendorf tubes and stored at −80 °C. Metagenomic DNA was extracted using a commercially available fecal DNA isolation kit (MP Biomedicals, LLC, Solon, OH, USA) according to the manufacturer’s protocol, with some modifications. DNA concentration and quality were assessed using a NanoDrop instrument and agarose gel electrophoresis, respectively. Sterile water was used as the negative control.

The V3–V4 regions of the bacterial *16S* rRNA gene were amplified with the universal primers, forward (5′-TCGTCGGCAGCGTCAGATGTGTATAAGAGACAG-3′) and reverse (5′-GTCTCGTGGGCTCGGAGATGTGTATAAGAGACAG-3′), combined with adapter sequences and barcode sequences. Two steps of PCR amplification were performed and the first round of PCR was operated using High-Fidelity DNA Polymerase (Solg 2X PCR smart mix) under the following thermal cycling conditions: denaturation at 95 °C for 3 min, followed by 25 cycles at 95 °C for 30s, 55 °C for 30s and 72 °C for 30s, with a final extension at 72 °C for 5 min. PCR products from the first step were purified using (Cosmo Genetech PCR purification Kit). The second round PCR was then performed under the following thermal cycling conditions: an initial denaturation at 95 °C for 3 min, followed by 8 cycles at 98 °C for 30 s, 55 °C for 30 s and 72 °C for 30s, and a final extension at 72 °C for 5 min. Finally, all the PCR products were sent to Macrogen (Seoul, South Korea) for sequencing.

### 2.11 Amplicon sequence variants (ASV) analysis

Illumina MiSeq raw data were processed to assign samples based on index sequences and to generate paired-end FASTQ files. Sequencing adapters and forward and reverse primers were removed using the Cutadapt (v3.2). The forward (Read1) and reverse (Read2) sequences were trimmed to 250 and 200 bp, respectively. Sequencing errors were corrected using the DADA2 package (v1.18.0) in R (v4.0.3), excluding paired-end reads with an expected error rate ≥2. Batch-specific error models were created to remove the technical noise. Paired-end reads were merged into single sequences, and the DADA2 consensus algorithm was used to identify and remove chimeric sequences. ASVs shorter than 350 bp were excluded from analysis.

### 2.12 Microbial community analysis

To normalize the data, subsampling was performed using the sample with the fewest reads as the reference, as implemented in QIIME (v1.9). Taxonomy assignment for each ASV was performed with BLAST+ (v2.9.0) against the NCBI 16S Microbial Database, with assignments made for sequences showing >85% query coverage and ≥85% sequence identity.

MAFFT (v7.475) was used for sequence alignment and a phylogenetic tree was constructed using FastTreeMP (v2.1.10). The alpha diversity was assessed using the Shannon index, Inverse Simpson index, rarefaction curves, and Chao1 estimates. The beta diversity was quantified using Weighted and Unweighted UniFrac distances. The relationships between samples were visualized using principal coordinate analysis and an Unweighted Pair Group Method with Arithmetic Mean (UPGMA) tree.

### 2.13 Bioinformatics analyses for the microbiome

Statistical analyses of the microbiome for LF, HF, HF + PCA, and HF + MPE were conducted using marker data profiling in MicrobiomeAnalyst 2.0 (Xia Lab, McGill University, Quebec, Canada). Total sum scaling was used for data normalization. Analyses at the phylum and genus levels were performed using abundance tables, and an interactive heatmap was generated using MicrobiomeAnalyst. The alpha diversity of the microbiome was calculated using the Chao1 and Shannon indices. Single-factor statistical comparisons were conducted using the analysis of variance (ANOVA) statistical method (*p*-values > 0.05). Correlation analyses between the microbiome and phenotypes of mice under LF, HF, HF + PCE and HF + MPE conditions were performed using OmicStudio (version 1.2, available at https://www.omicstudio.cn).

### 2.14 Metabolite analysis using ultra-performance liquid chromatography-quadrupole time-of-flight mass spectrometry (UPLC-Q-TOF MS)

Metabolites were extracted from the dried seaweed samples using 70% aqueous methanol containing an internal standard (IS; terfenadine for the positive mode or zidovudine for the negative mode) using a bullet blender (Next Advance, Troy, NY, USA). After centrifugation (14,000 rpm × 10 min at 4 °C), the supernatants were analyzed using UPLC-Q-TOF MS (Xevo G2-S, Waters, Milford, MA, USA) equipped with an Acquity UPLC BEH C18 column (2.1 mm × 100 mm, 1.7 μm; Waters) at 40 °C. The flow rate was set at 0.35 mL/min and metabolites were eluted using gradients of water containing 0.1% formic acid (solvent A) and acetonitrile (solvent B) as follows: 0 min 0% B, 1 min 0% B, 9 min 100% B, 10 min 100% B, 11 min 0% B. The Q-TOF MS system detected the eluted metabolites in positive or negative electrospray ionization mode with a scan range from 50 to 1,500 m/z at a scan time of 0.2 s. The optimal conditions of the TOF-MS system were 800 L/h desolvation gas flow rate, 400 °C desolvation temperature, 100 °C ion source temperature, 3 kV of capillary, and sampling cone voltage of 40 V. Leucine-enkephalin ([M + H] = 556.2771, [M – H] = 554.2615) was used as a lock mass reference. Quality control samples containing a mixture of all samples were analyzed for each set. MS/MS spectra were obtained using a collision energy ramp from 10 to 30 eV or 20 to 40 eV. The MS dataset analyzed using UPLC-Q-TOF MS was collected, aligned using MarkerLynx software (Waters), and normalized to the IS. The metabolites were tentatively identified based on MS/MS spectra using online databases (ChemSpider database in UNIFI, METLIN database,^1^ and human metabolome databases.^2^

Partial least-squares discriminant analysis (PLS-DA) score plots of PCE and MPE’s metabolites analyzed using GC/MS, UPLC-Q-TOF MS, and HPLC and their qualify parameters. The quality of the PLS-DA model was evaluated using *R*^2^*X* = 0.811, *R*^2^*Y* = 0.999, and *Q*^2^ = 0.995, and *p*-value (2.33 × 10^−5^), with validation through 200-permutation tests (thresholds: *R*^2^ intercept < 0.5, *Q*^2^ intercept < −0.5). Variable importance in projection (VIP) scores and univariate *p*-values was calculated to identify key differential metabolites between PCE and MPE. The two seaweed samples (PCE and MPE) were distinctly separated along the first component (*t*[1]). Metabolites with VIP scores > 0.87 and *p* < 0.05 were considered statistically significant and are listed in Supplementary Table S3 and Supplementary Figure 1.

### 2.15 Statistical analysis

All data are presented as the mean ± standard error of the mean (SEM). Statistical analyses were conducted using one-way analysis of variance (ANOVA), followed by Bonferroni’s or Duncan’s *post hoc* multiple comparison tests as appropriate. A *p*-value < 0.05 was considered statistically significant. Spearman’s rank correlation coefficients were calculated to assess associations between metabolite levels and metabolic parameters. The resulting correlation matrix was visualized as a heatmap with a color gradient scale indicating the strength and direction of correlations. All statistical analyses and graph generation were performed using GraphPad Prism (versions 9.0.2 and 11.0; GraphPad Software, San Diego, CA, USA) and SPSS version 24.0 (SPSS Inc., Chicago, IL, USA).

## 3 Results

### 3.1 Phenolic profiles, antioxidant activities, and residual salinity of PCE and MPE

To address the essential compositional context, we quantified the phenolic and flavonoid contents, antioxidant capacities, and residual salinity of PCE and MPE (Tables 1, 2). Polyphenols and flavonoids are well-recognized contributors to the antioxidant and metabolic effects of seaweed extracts, influencing oxidative stress and inflammation that underlie obesity-related metabolic dysfunction (27, 28). PCE exhibited a significantly higher total phenolic content than MPE (72.48 ± 1.54 vs. 19.94 ± 0.77 mg GAE/g dry extract), whereas MPE contained a comparatively higher flavonoid content. Consistent with these compositional differences, PCE demonstrated stronger radical-scavenging activity, as evidenced by lower IC₅₀ values in both DPPH and ABTS assays relative to MPE (Table 1), supporting its greater antioxidant potential.

**Table 1:** Total phenolic content (TPC), total flavonoid content (TFC), and antioxidant activities of Peyssonnelia caulifera (PCE) and Meristotheca papulose (MPE).

Parameter	PCE	MPE
TPC (mg GAE/g dry extract)	72.48 ± 1.54	19.94 ± 0.77
TFC (mg GAE/g dry extract)	0.80 ± 2.20	1.87 ± 3.57
DPPH (IC₅₀)	16.09	30.09
ABTS (IC₅₀)	11.03	31.42

ABTS, 2,2′-azino-bis-(3-ethylbenzothiaziline-6-sulfonic acid) diammonium salt; DPPH, 2-diphenyl-1-picrylhydrazyl; GAE, gallic acid equivalent.

**Table 2:** Salt content of PCE and MPE.

Extract	Solution concentration (mg/mL)	Salinity reading (% w/v)	NaCl-eq (mg/g extract)
PCE	0.5	ND (<0.01)	<100
MPE	0.5	ND (<0.01)	<100

NaCl-eq, NaCl-equivalent; ND, indicates below the limit of detection (LOD = 0.01% w/v).

To evaluate residual inorganic salts that may remain after ethanol extraction-a factor that can influence metabolic and physiological outcomes in animal studies (29)—we measured salinity at a solution concentration of 0.5 mg/mL Both PCE and MPE showed salinity below the detection limit (<0.01% w/v; <100 mg NaCl-equivalent/g extract) (Table 2). These results indicate that both extracts contained relatively low or no residual salt levels, suggesting that the observed metabolic effects are unlikely to be driven by inorganic salts and are more plausibly attributable to differences in their bioactive composition.

### 3.2 PCE and MPE supplementation ameliorated HFD-induced metabolic parameters

**Figure 1:**
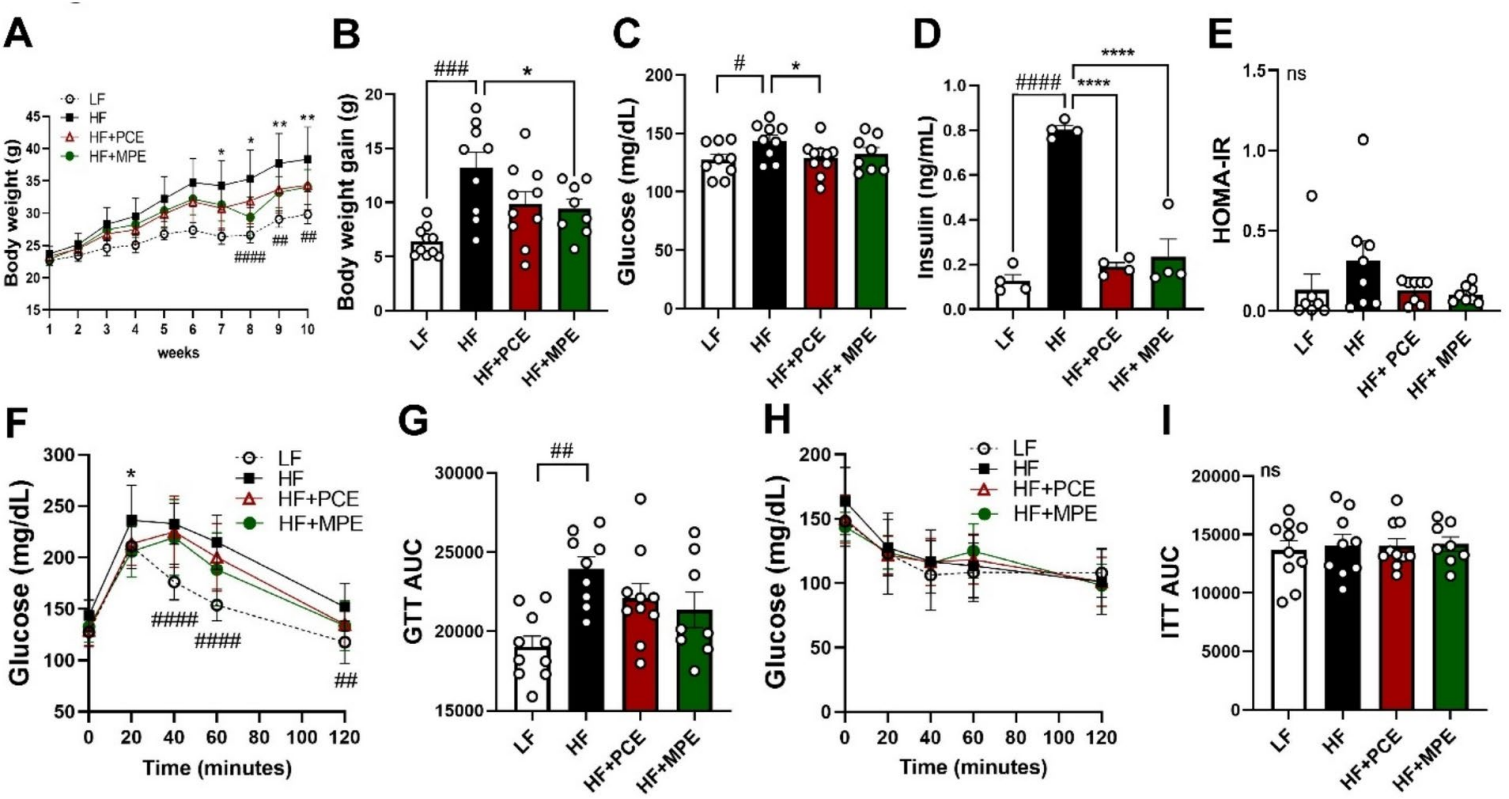
PCE and MPE partly ameliorated HFD-mediated abnormal glucose metabolism. Male C57BL/6 mice were fed with LF (white circle and bar), HF (black square and bar) or HFD with daily oral of either PCE (10 mg/kg BW, HF + PCE, red triangle and bar) or MPE (10 mg/kg BW, HF + MPE, green circle and bar) for 8 weeks (*n* = 8–10 per group). **(A)** Body weight (g). **(B)** Body weight gain (g). **(C)** Fasting glucose level (mg/dL). **(D)** Insulin level in serum (ng/mL). **(E)** HOMA-IR. **(F)** Glucose tolerance test (GTT). **(G)** The area under the curve (AUC) during GTT. **(H)** Insulin tolerance test (ITT). **(I)** AUC during ITT. Data are expressed as the mean ± SEM (*n* = 8–10/group). Ns represents no significance. Bars with different letters are significantly different according to one-way ANOVA with Bonferroni’s comparison test; ^#^*p* < 0.05, ^##^*p* < 0.01, ^###^*p* < 0.001, ^####^*p* < 0.0001 (LF vs. HF), ^*^p < 0.05, ^**^p < 0.01, ^****^p < 0.0001 (HF vs. HF + PCE or MPE) by one-way ANOVA with Bonferroni’s comparison test or student *t*-test. BW, body weight; HF, high-fat; HFD, high-fat diet; HOMA-IR, homeostatic model assessment for insulin resistance; LF, low-fat; MPE, *Meristotheca papulosa* extract; PCE, *Peyssonnelia caulifera Okamura* extract.

To investigate the effects of PCE and MPE supplementation on obesity in the HFD-induced obese mouse model, changes in BW, food intake, and energy efficiency were monitored over an 8-week period. At baseline, the average BW was approximately 23.1 ± 0.16 g. After 8 weeks of dietary intervention, the final weights were recorded as 29.82 ± 0.47 g for the LF group, 38.31 ± 1.67 g for the HF group, 34.33 ± 1.22 g for the HF + PCE group, and 34.03 ± 0.95 g for the HF + MPE group (Figure 1A; Supplementary Table S4). Notably, mice in the HF + MPE group exhibited a significant reduction in weight gain compared with those in the HF group, whereas the HF + PCE group demonstrated a decreasing trend without statistical significance (Figure 1B). Given the potential influence of food intake on BW gain, the dietary and caloric intakes were analyzed. Average dietary intake was 4.65 ± 0.26 g for the LF group, 2.38 ± 0.12 g for the HF group, 2.23 ± 0.13 g for the HF + PCE group, and 2.32 ± 0.16 g for the HF + MPE group. While the HF-fed groups (HF, HF + PCE, and HF + MPE) consumed less food than the LF group, the differences between the HF, HF + PCE, and HF + MPE groups were not significant. Energy efficiency, calculated as the ratio of weight change to dietary intake, was significantly lower in the HF + MPE group than in the HF group (Supplementary Table S4).

To assess the effects of PCE and MPE on glucose regulation, fasting glucose levels were measured after a 12-h fast. HFD consumption significantly elevated fasting glucose levels; however, PCE supplementation mitigated this increase, whereas MPE supplementation did not exhibit a similar effect (Figure 1C). The HFD improved insulin levels in the HF + PCE and HF + MPE groups (Figure 1D). Both the PCE and MPE treatments demonstrated a trend toward reduced HOMA-IR values compared with that of the HF group, although these differences were not statistically significant (Figure 1E). During the GTT, PCE and MPE supplementation enhanced the glucose disposal rate relative to the HF group; however, these effects were not statistically significant (Figures 1F,G). Similarly, the ITT results indicated comparable trends (Figures 1H,I) Collectively, suggesting that PCE and MPE supplementation may contribute to enhanced glucose tolerance, improved insulin sensitivity, and better energy homeostasis in the context of HFD-induced metabolic disturbances.

### 3.3 PCE and MPE supplementation attenuated HFD-induced adipocyte hypertrophy and inflammation

**Figure 2:**
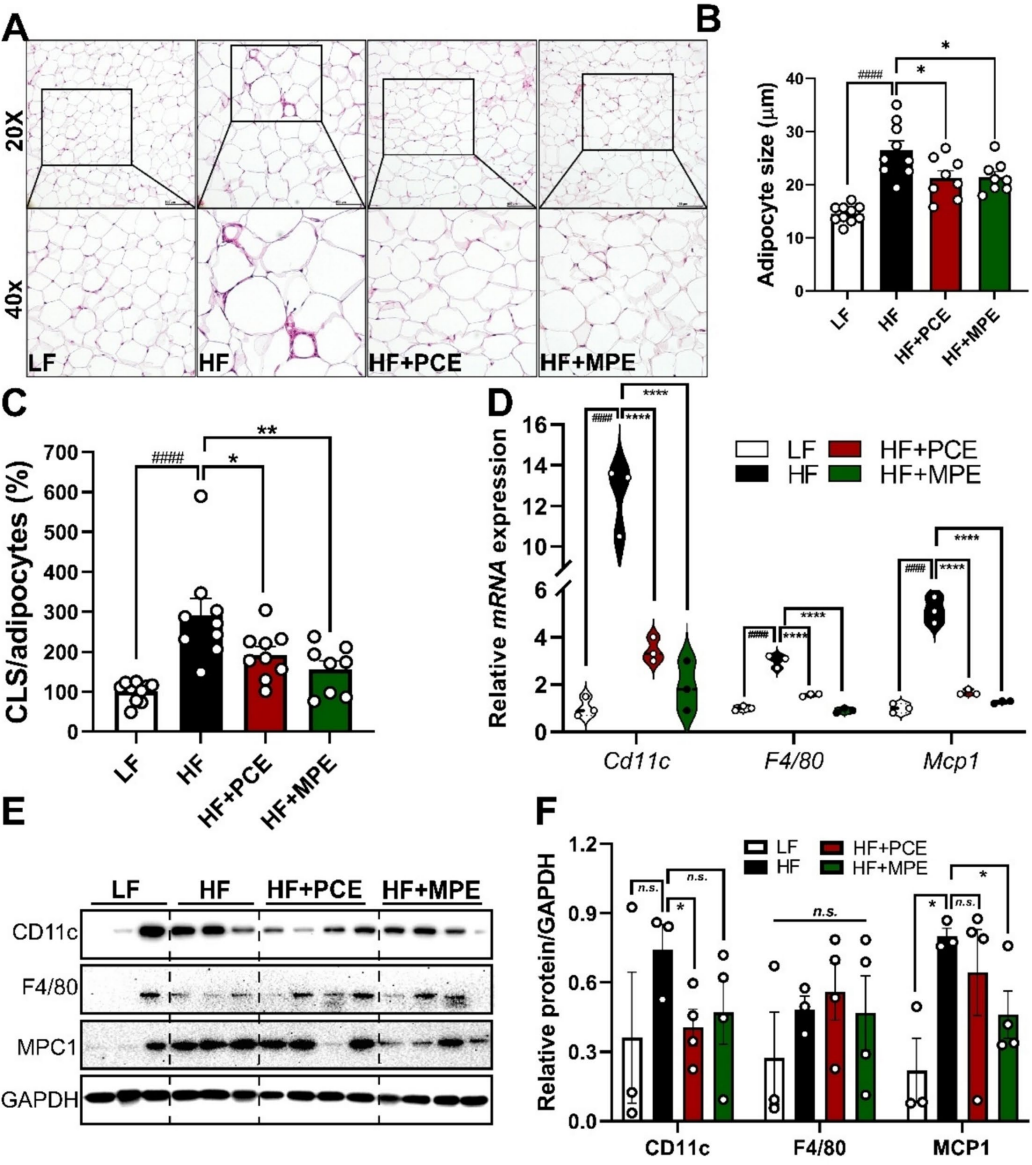
PCE and MPE supplementation attenuated HF-induced adipocyte hypertrophy and inflammation. Male C57BL/6 mice were fed with LF (white bar), HF (black bar), or HFD with daily oral of either PCE (10 mg/kg BW, HF + PCE, red bar) or MPE (10 mg/kg BW, HF + MPE, green bar) for 8 weeks (*n* = 8–10 per group). **(A)** H&E staining of adipose tissue (20× and 40 magnification). **(B)** Adipocyte size (M). **(C)** Crown-like structures (CLS) numbers/adipocytes (%) in epididymal adipose tissue. **(D)** mRNA expression of *Cd11c*, *F4/80,* and *Mcp1* in epididymal adipose tissue determined using real-time PCR. **(E)** Representative Western blot bands of CD11c, F4/80, MCP1, and GAPDH in epididymal adipose tissue. **(F)** Quantification of protein expression normalized to GAPDH. Data are expressed as the mean ± SEM (*n* = 8–10/group). s represents no significance. Bars with different letters are significantly different according to one-way ANOVA with Bonferroni’s comparison test; ^####^*p* < 0.0001 (LF vs. HF), ^*^p < 0.05, ^**^p < 0.01, ^****^p < 0.0001 (HF vs. HF + PCE or MPE) by one-way ANOVA with Bonferroni’s comparison test or student *t*-test. BW, body weight; H&E, hematoxylin and eosin; HF, high-fat; HFD, high-fat diet; LF, low-fat; MPE, *Meristotheca papulosa* extract; PCE, *Peyssonnelia caulifera Okamura* extract.

Next, we investigated the impact of PCE and MPE supplementation on adipose tissue remodeling and inflammation. Obesity, characterized by excessive fat accumulation, was evaluated by measuring the adipose tissue weight. The epididymal fat (visceral fat) weight was significantly higher in the HF group than in the LF group. However, no significant differences were observed between the HF, HF + PCE, and HF + MPE groups (Supplementary Table S4). Morphological alterations in visceral fat were assessed using H&E staining of epididymal fat, which revealed smaller adipocytes in the HF + PCE and HF + MPE groups than those in the HF group (Figure 2A). Quantitative analysis of adipocyte size and crown-like structure (CLS) formation demonstrated a significant increase in both parameters in the HF group compared with those in the LF group. Notably, the adipocyte size (Figures 2A,B) and CLS (Figure 2C) were significantly reduced in the HF + PCE and HF + MPE groups compared with those in the HF group. Adipose tissue inflammation, exacerbated by excessive fat deposition, is triggered by immune cell infiltration, primarily by macrophages, leading to elevated secretion of inflammatory cytokines. Consistent with adipocyte size and CLS (Figures 2A–C), genes associated with adipose inflammation, including *Cd11c*, *F4/80*, and *Mcp1*, were significantly upregulated in the HF group compared with those in the LF group. Notably, PCE and MPE treatment attenuated the expression of these inflammatory markers (Figure 2D). Additionally, HF-fed mice exhibited increased inflammatory protein expression, whereas PCE and MPE significantly attenuated CD11c and MCP1 protein levels, respectively (Figures 2E,F). These findings underscore the efficacy of PCE and MPE in ameliorating adipocyte hypertrophy and mitigating adipose inflammation, highlighting their potential to counteract HFD-induced metabolic perturbations.

### 3.4 PCE and MPE supplementation reduced HFD-induced hepatic lipid accumulation and liver damage

**Figure 3:**
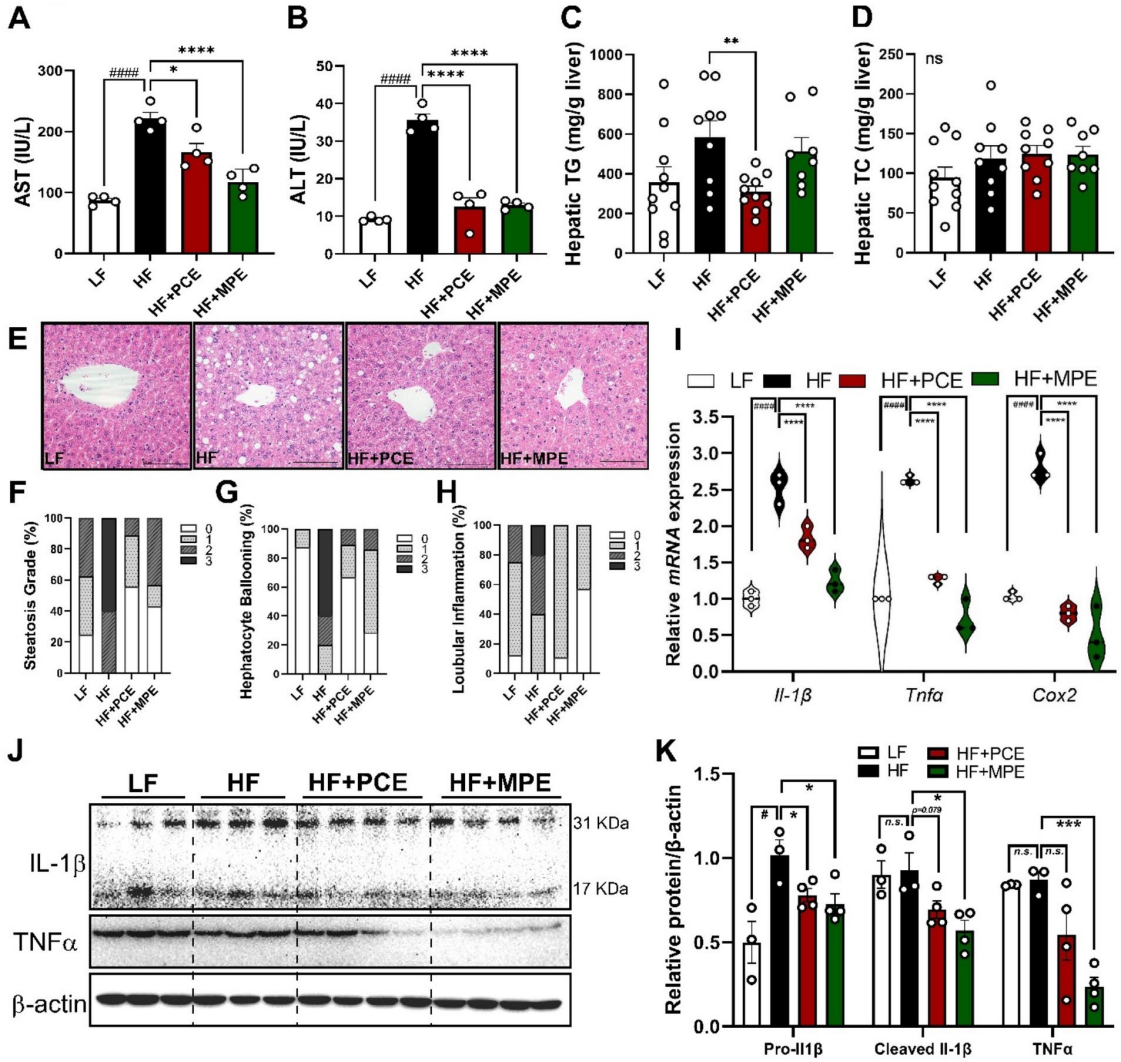
PCE or MPE supplementation reduced HFD-induced hepatic lipid accumulation and inflammation. Male C57BL/6 mice were fed with LF (white bar), HF (black bar) or HFD with daily oral of either PCE (10 mg/kg BW, HF + PCE, red bar) or MPE (10 mg/kg BW, HF + MPE, green bar) for 8 weeks (*n* = 8–10 per group). **(A)** Plasma levels of aspartate transaminase levels (AST, IU/L). **(B)** Plasma levels or alanine transaminase levels (ALT, IU/L). **(C)** Hepatic TG content (mg/g liver weight). **(D)** Hepatic TC content (mg/g liver weight). **(E)** Hematoxylin and eosin (H&E) staining of liver tissue (40× magnification). **(F)** Histological score of hepatic steatosis grade (%). **(G)** Histological score of hepatocyte ballooning (%). **(H)** Histological score of lobular inflammation (%). **(I)** Hepatic mRNA expression of interleukin 1 beta (Il-1β), tumor necrosis factor alpha (Tnf*α*), and cyclooxygenase 2 (Cox2) using qPCR. **(J)** Representative Western blot bands of pro-IL-1β, TNFα, and GAPDH in liver tissue. **(K)** Quantification of protein expression normalized to GAPDH. Data are expressed as the mean ± SEM (*n* = 8–10/group). represents no significance. Bars with different letters are significantly different according to one-way ANOVA with Bonferroni’s comparison test; ^####^*p* < 0.0001 (LF vs. HF), ^*^p < 0.05, ^**^p < 0.01, ^****^p < 0.0001 (HF vs. HF + PCE or MPE) by one-way ANOVA with Bonferroni’s comparison test or student *t*-test. BW, body weight; H&E, hematoxylin and eosin; HF, high-fat; HFD, high-fat diet; LF, low-fat; MPE, *Meristotheca papulosa* extract; PCE, *Peyssonnelia caulifera Okamura* extract; TC, total cholesterol; TG, triglyceride.

To evaluate the protective effects of PCE and MPE against HFD-induced hepatic steatosis and inflammation, we conducted H&E staining of liver tissues and measured key biomarkers of liver damage. The serum levels of AST and ALT, widely recognized indicators of liver injury, were assessed. The HF group displayed significantly elevated plasma AST and ALT levels compared with those of the LF group, which were notably reduced by both PCE and MPE treatments (Figures 3A,B). Despite the lack of significant differences in liver tissue weight among the groups (Supplementary Table S4), H&E staining revealed prominent intrahepatic lipid accumulation in the HF group, characterized by lipid droplet formation (Figure 3E). Notably, hepatic TG levels, which were significantly increased in the HF group, were effectively reduced by PCE but not by MPE treatment (Figure 3C). Hepatic TC levels remained unchanged across all groups (Figure 3D). Histological evaluation further confirmed the protective effects of PCE and MPE against HFD-induced hepatic injury. The increased histological scores for steatosis observed in the HF group were markedly reduced in the HF + PCE and HF + MPE groups (Figure 3F). Similarly, hepatocyte ballooning and lobular inflammation, two critical markers of hepatic damage, exhibited a significant downward trend in the HF + PCE and HF + MPE groups compared with those in the HF group (Figures 3G,H). In addition to histological improvements, the expression of intrahepatic inflammatory genes, including interleukin-1 beta (*Il-1β*), tumor necrosis factor-alpha (*Tnf-α*), and cyclooxygenase-2 (*Cox2*), was substantially reduced in the HF + PCE and HF + MPE groups relative to the HF group (Figure 3I). Correspondingly, HF significantly increased pro-IL-1β protein levels compared with the LF group, which was significantly alleviated by PCE and MPE. In addition, PCE partially reduced cleaved IL-1β levels (*p* = 0.079), while MPE treatment markedly decreased cleaved IL-1β and TNF-α protein expression, as confirmed by quantitative protein analysis (Figures 3J,K). These findings indicated that PCE and MPE supplementation effectively mitigated hepatic steatosis, inflammation, and the associated damage induced by HFD consumption.

### 3.5 PCE and MPE improve HFD-induced intestinal epithelial permeability and alter tight junction protein expression

**Figure 4:**
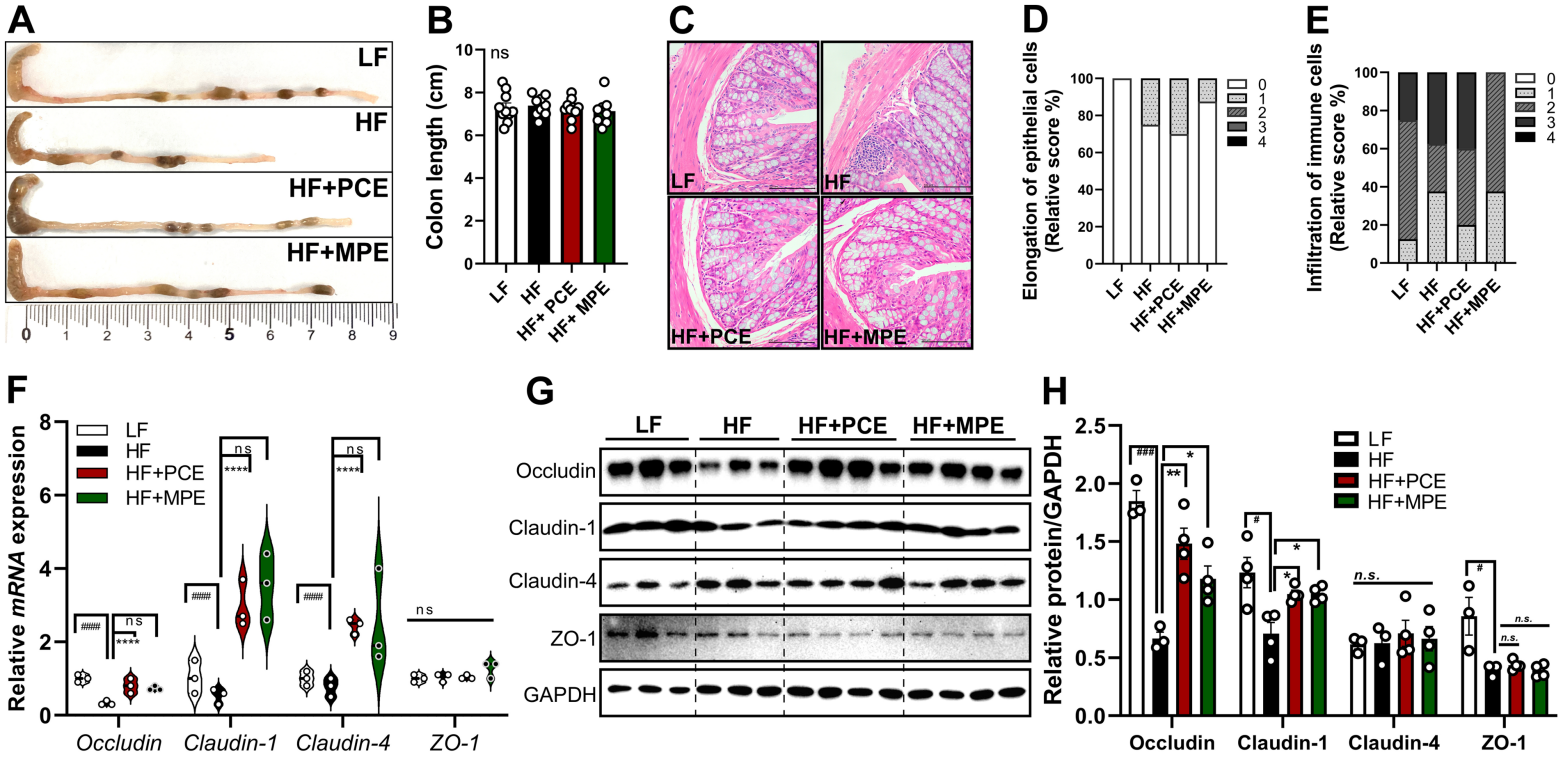
PCE and MPE improve HFD-induced intestinal epithelial permeability through modulation of tight junction proteins. Male C57BL/6 mice were fed with LF (white bar), HF (black bar), or HFD with daily oral of either PCE (10 mg/kg BW, HF + PCE, red bar) or MPE (10 mg/kg BW, HF + MPE, green bar) for 8 weeks (*n* = 8–10 per group). **(A)** Representative images of the colon of mice. **(B)** Quantified colon length (CM). **(C)** Staining colon tissues of mice with hematoxylin and eosin (H&E) (20× magnification). **(D)** Histological score of the colons for infiltration of immune cells (%). **(E)** Histological score of the colons for elongation of epithelial cells (%). **(F)** Gene expression of tight junction proteins, including *occludin*, *claudin-1*, *claudin-4,* and *ZO-1*. Data are expressed as the mean ± SEM (*n* = 8–10/group). **(G)** Representative Western blot bands of occludin, claudin-1, claudin-4, ZO-1, and GAPDH in colon tissue. **(H)** Quantification of protein expression normalized to GAPDH. ns represents no significance. Bars with different letters are significantly different according to one-way ANOVA with Bonferroni’s comparison test; ^####^*p* < 0.0001 (LF vs. HF), ****p < 0.0001 (HF vs. HF + PCE or MPE) by one-way ANOVA with Bonferroni’s comparison test or student *t*-test. BW, body weight; H&E, hematoxylin and eosin; HF, high-fat; HFD, high-fat diet; LF, low-fat; MPE, *Meristotheca papulosa* extract; PCE, *Peyssonnelia caulifera Okamura* extract.

Excessive fat intake is associated with inflammation and structural changes in the colon, including a reduction in overall colon length (30). Unexpectedly, there were no significant differences in the colon length between the groups (Figures 4A,B). Inflammatory infiltration of the colonic mucosa, a hallmark of HFD-induced intestinal damage (31), was less pronounced in the HF + MPE group than that in the HF group. However, the inflammatory cell infiltration scores in the HF + PCE group were comparable to those in the HF group (Figures 4C,D). Similarly, the elongation of epithelial cells was slightly reduced in the HF + MPE group, but not in the HF + PCE group, compared with those in the HF group (Figures 4C,E).

Tight junction proteins, which are essential for maintaining intestinal barrier integrity and gut immune function, are downregulated by the chronic intake of excessive HFDs (9). Intestinal permeability was assessed by analyzing the expression of key tight junction proteins, including *occludin*, *claudin-1*, *claudin-4*, and *zonula occludens-1* (*Zo-1*). PCE treatment significantly augmented the expression of tight junction proteins that were diminished by the HFD. The MPE treatment exhibited a similar trend, but the changes were not statistically significant. *Zo-1* expression did not considerably differ between groups (Figure 4F). Consistent with these findings, HF group significantly reduced the protein expression of Occludin, Claudin-1, and ZO-1 compared with LF group. Both PCE and MPE treatments markedly upregulated Occludin levels, while MPE treatment alone partially increased claudin-1 expression compared with the HF group (*p* = 0.082) (Figures 4G,H). These findings suggest that the increased gut permeability induced by an HFD, characterized by reduced tight junction protein expression, may facilitate the translocation of gut bacteria or microbial byproducts into the systemic circulation. This process could contribute to the inflammation observed in the liver and adipose tissues. PCE and MPE appeared to mitigate these effects by improving intestinal barrier integrity and modulating tight junction protein expression, with PCE showing a more pronounced effect.

### 3.6 PCE and MPE modulate gut microbiota composition and enhance intestinal barrier function in an HFD model

**Figure 5:**
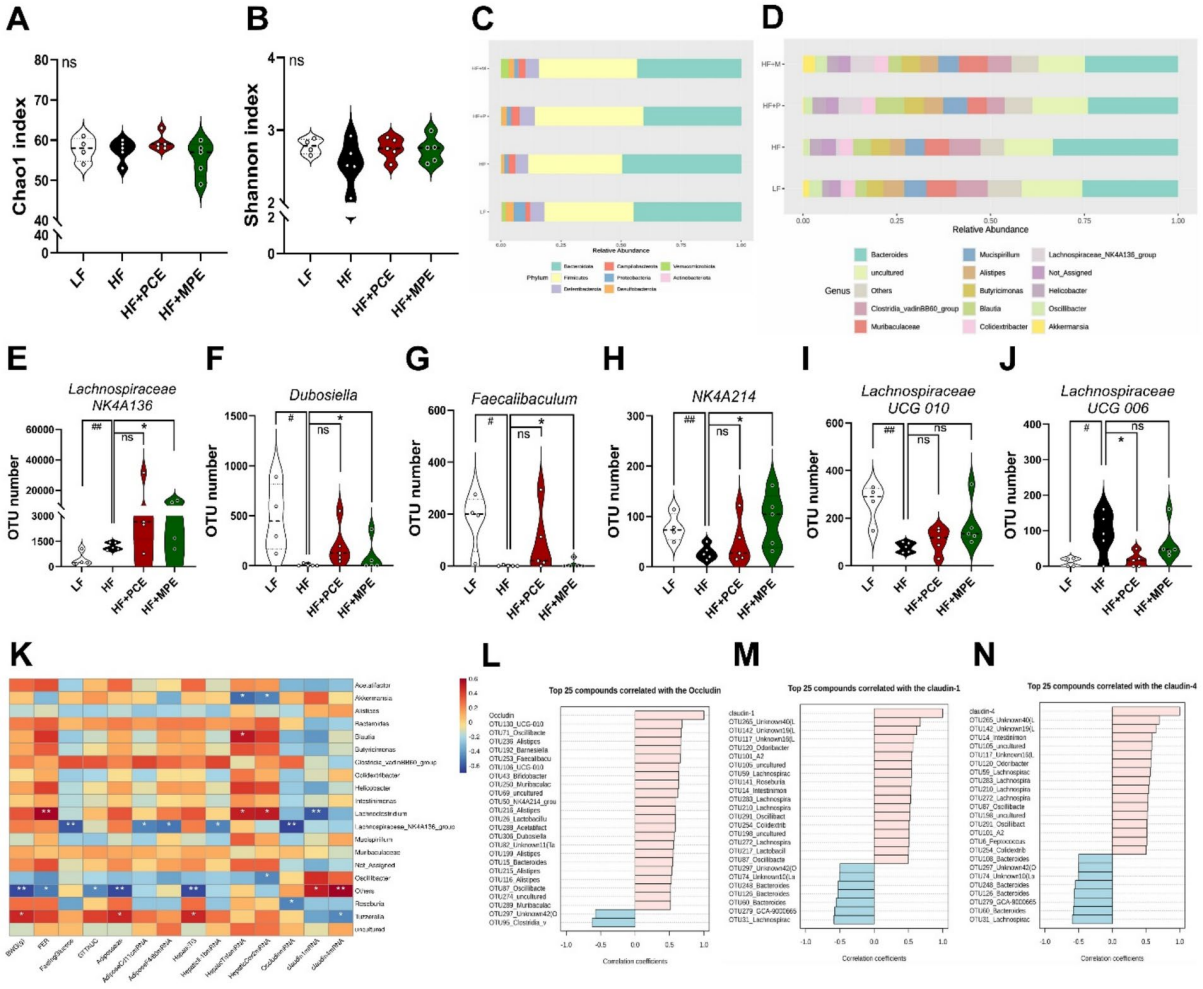
PCE and MPE supplementation alters gut microbiota composition in HFD-fed mice. **(A,B)** OTU number, Shannon index, relative abundance using α diversity analysis. **(C,D)** Contribution of the phyla and genera in the four groups. Average relative abundance of detected bacterial phyla: **(E)** *Lachnospiraceae NK4A136*, **(F)** *Dubosiella*, **(G)** *Faecalibaculum*, **(H)** *NK4A214*, **(I)** *Lachnospiraceae UCG-010*, **(J)** *Lachnospiraceae UCG-006*. **(K)** Correlation heatmap analysis of the dominant gut microbiota (top 20 genera) with obesity-associated metabolic parameters and intestinal homeostasis indexes. Red represents a positive correlation, and blue represents a negative correlation. Correlation analysis between the abundance of taxa (at genus level) and tight junction mRNA expression, **(L)** occludin, **(M)** claudin-1, **(N)** claudin-4. Data are expressed as the mean ± SEM. ns represents no significance. Bars with different letters are significantly different according to one-way ANOVA with Bonferroni or Duncan’s comparison test; ^#^*p* < 0.05, ^##^*p* < 0.01 (LF vs. HF), ^*^p < 0.05 (HF vs. HF + PCE or MPE) by one-way ANOVA with Bonferroni or Duncan’s comparison test or student *t*-test. HF, high-fat; HFD, high-fat diet; LF, low-fat; MPE, *Meristotheca papulosa* extract; PCE, *Peyssonnelia caulifera Okamura* extract.

We investigated the impact of PCE and MPE treatments on the gut microbiome composition in mice fed an HFD. Fecal microbiota from each experimental group were analyzed to evaluate microbial diversity using *α*-diversity indices, including the Chao1 index for microbial richness and Shannon index for overall diversity. The results demonstrated high microbial richness across all experimental groups, as indicated by the Chao1 index (Figure 5A). The Shannon index revealed that the LF group had the highest microbial diversity, whereas the HF group had the lowest. However, no significant differences were observed between the groups (Figure 5B). To examine the differences in the gut microbiota due to extract treatment, we performed abundance profiling at both the phylum and genus levels (Figures 5C,D). At the phylum level (Figure 5C), the HF group showed a higher abundance of *Bacteroides* than that in the LF, HF + PCE, and HF + MPE groups, whereas the LF and HF + MPE groups exhibited an increase in *Verrucomicrobiota.* At the genus level (Figure 5D), the HF + PCE and HF + MPE groups showed a reduced abundance of *Bacteroides* compared with that in the HF group, whereas the *Lachnospiraceae NK4A136* group showed an increase in abundance. Further genus-specific analyses (Figures 5E–H) revealed significant increases in *Lachnospiraceae NK4A136, Dubosiella, Faecalibaculum*, and *Ruminococcaceae NK4A214* in the MPE-treated group. Although *Lachnospiraceae UCG-010* abundance also increased in the MPE group, this change was not statistically significant (Figure 5I). These genera also showed increased abundance in the PCE group; however, the differences were not statistically significant. Additionally, *Lachnospiraceae UCG-006*, which is typically enriched in HFD-fed mice, was notably reduced following PCE treatment (Figure 5J).

To assess the impact of PCE and MPE on the gut microbiota composition and intestinal barrier function, we performed a correlation analysis between bacterial genera and metabolic parameters (Figure 5K). Significant correlations are indicated by asterisks (*p* < 0.05, *p* < 0.01). *Akkermansia* negatively correlated with hepatic *Tnfα* and *Cox2* mRNA (^*^p < 0.05). *Blautia* postively correlated with hepatic *Tnfα* mRNA (^*^p < 0.05). *Lachnoclostridium* postively correlated with FER (^**^p < 0.01), hepatic *Tnfα,* and *Cox2 mRNA* (^*^p < 0.05); and *Lachnoclostridium* negatively correlated with claudin1 mRNA (^**^p < 0.01). *Lachnospiraceae NK4A136* negatively correlated with fasting glucose (*^*^p < 0.01), adipose *Cd11c, F4/80* mRNA (^*^p < 0.05), hepatic *Il-1β* mRNA (^*^p < 0.05), and *occludin* mRNA (*^*^p < 0.01). *Oscillibacter* negatively correlated with hepatic *Cox2* mRNA levels (^*^p < 0.05). *Tuzzerella* positively correlated with BWG, adipose size, and hepatic TG (^*^p < 0.05) and negatively correlated with claudin4 mRNA. LPS-producing bacteria in HFDs impair intestinal barrier function by downregulating tight junction proteins, such as ZO-1 and occludin (32). Moreover, *Lachnospiraceae* are known to reinforce the intestinal barrier and mitigate metabolic endotoxemia by producing butyrate (33). Consistent with this, our findings showed positive correlations between *Claudin-1* and *Claudin-4* mRNA expression, and *Lachnospiraceae* abundance, whereas *Bacteroides* exhibited a negative correlation (Figures 5L–N).

### 3.7 PCE exhibits greater potential than MPE in reducing LPS-induced inflammation and adipogenesis *in vitro*

**Figure 6:**
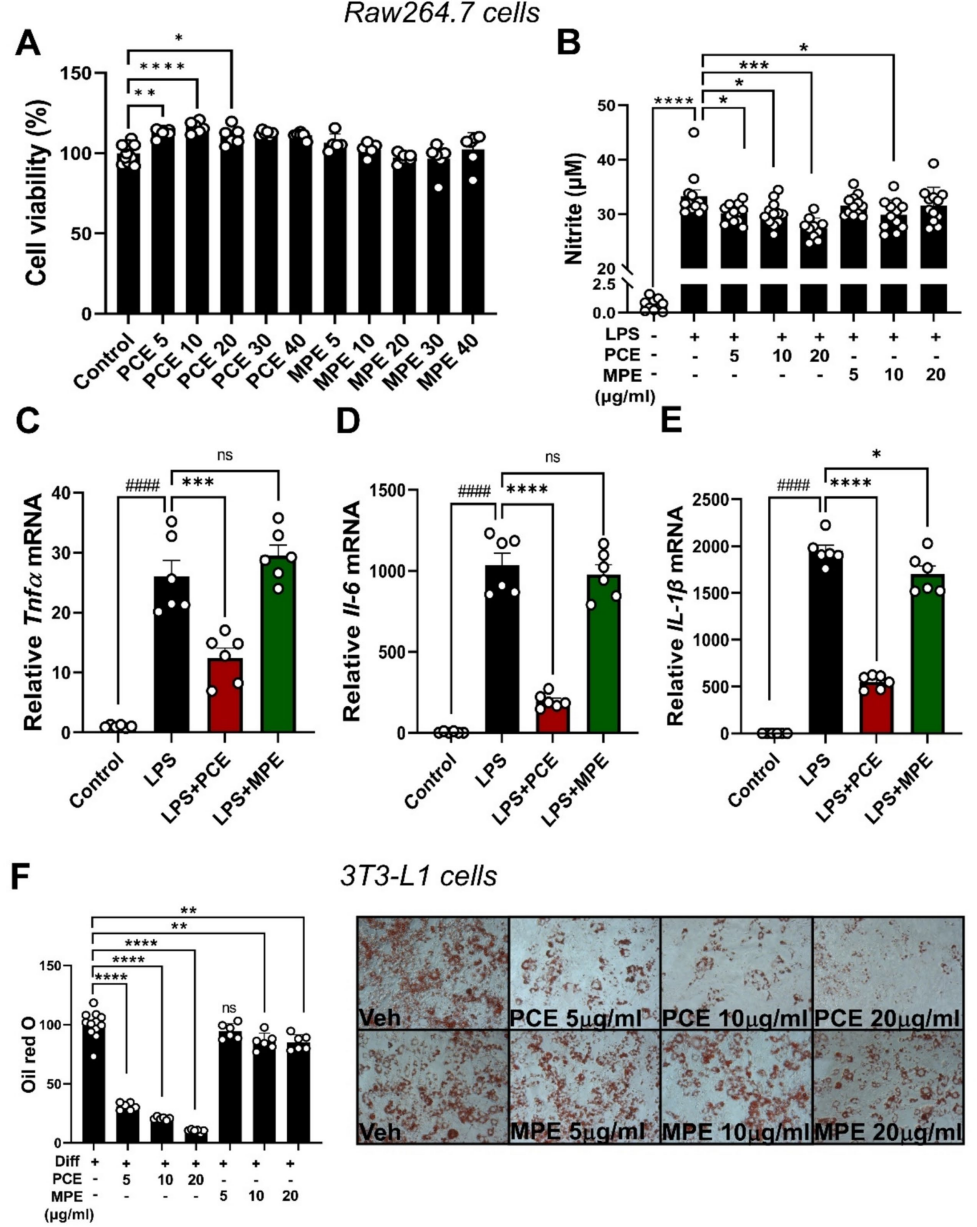
PCE reduced NO and inflammation in LPS-treated Raw264.7 cell and adipogenesis in 3T3-L1 adipocytes. **(A)** Cell viability of Raw264.7 cells after treatment with compounds with or without PCE or MPE (5–40 μg/mL) for 24 h. Raw264.7 cells (1 × 10^5^ cells/well) were pretreated with various PCE and MPE concentrations (5–29 μg/mL) for 48 h and then starved in DMEM for 12–18 h before LPS stimulation (1 μg/mL) for 24 h in 1% FBS-containing medium with or without PCE and MPE treatment. **(B)** NO production using Griess reagent. **(C–E)** Real-time PCR for inflammatory cytokines genes, *TNF-α, IL-6,* and *IL-1β*. The 3 T3-L1 cells were seeded and induced to differentiation in the presence of DMSO (vehicle control), PCE (5–20 μg/mL) or MPE (5–20 μg/mL) for 7 days. **(F)** TG accumulation was visualized by Oil red O staining and representative images from three separate experiments are shown. Data are expressed as the mean ± SEM. ns indicates no significance. Bars with different letters are significantly different according to one-way ANOVA with Bonferroni’s *post hoc* test. ^####^*p* < 0.0001 (Veh vs. LPS), ^*^p < 0.05, ^**^p < 0.01, ^***^p < 0.001, ^****^p < 0.0001 (CON, LPS, or Veh vs. PCE or MPE-treatment) by one-way ANOVA with Bonferroni’s comparison test or student *t*-test. HF, high-fat; HFD, high-fat diet; LF, low-fat; LPS, lipopolysaccharide; MPE, *Meristotheca papulosa* extract; NO, nitric oxide; PCE, *Peyssonnelia caulifera Okamura* extract; TG, triglyceride.

To evaluate the anti-inflammatory properties of PCE and MPE in the context of HFD-induced inflammation, we conducted *in vitro* experiments using LPS-stimulated RAW264.7 macrophages. Initial cell viability assays confirmed that both PCE and MPE were non-cytotoxic at concentrations up to 40 μg/mL (Figure 6A). In LPS-stimulated RAW264.7 macrophages, treatment with 10 μg/mL of PCE or MPE significantly attenuated nitric oxide production (Figure 6B). Based on these results, used 10 μg/mL of PCE and MPE was used in subsequent experiments. Notably, PCE demonstrated a distinct ability to further suppress pro-inflammatory cytokine expression, specifically *Tnfα* and *Il-6* mRNA levels (Figures 6C,D). Both PCE and MPE mitigated LPS-induced upregulation of *Il-1β* mRNA expression, with comparable efficacy (Figure 6E). PCE showed greater downregulation of pro-inflammatory mRNA expressions (Figures 6C–E). This selective downregulation of key cytokines by PCE underscores its potential for more comprehensive interference with macrophage-mediated inflammatory pathways compared to that with MPE treatment. We further examined the anti-adipogenic properties of PCE and MPE *in vitro* using 3T3-L1 adipocytes. Consistent with the anti-inflammatory responses observed following PCE treatment, lipid accumulation during adipogenesis was significantly reduced by PCE treatment in a dose-dependent manner, as demonstrated using Oil Red O (ORO) staining. In contrast, MPE treatment showed comparatively limited effects on adipogenesis, although it significantly reduced ORO (Figure 6F). These findings suggest that, while both PCE and MPE exhibit anti-inflammatory and anti-adipogenic properties, PCE demonstrates a more robust ability to suppress LPS-induced inflammatory responses and reduce fat accumulation *in vitro*. This highlights its potential as an effective intervention for the inflammation and adipogenesis associated with HFD-induced metabolic disorders.

### 3.8 Differential metabolite profiles of PCE and MPE analyzed using UPLC-QTOF MS

**Figure 7:**
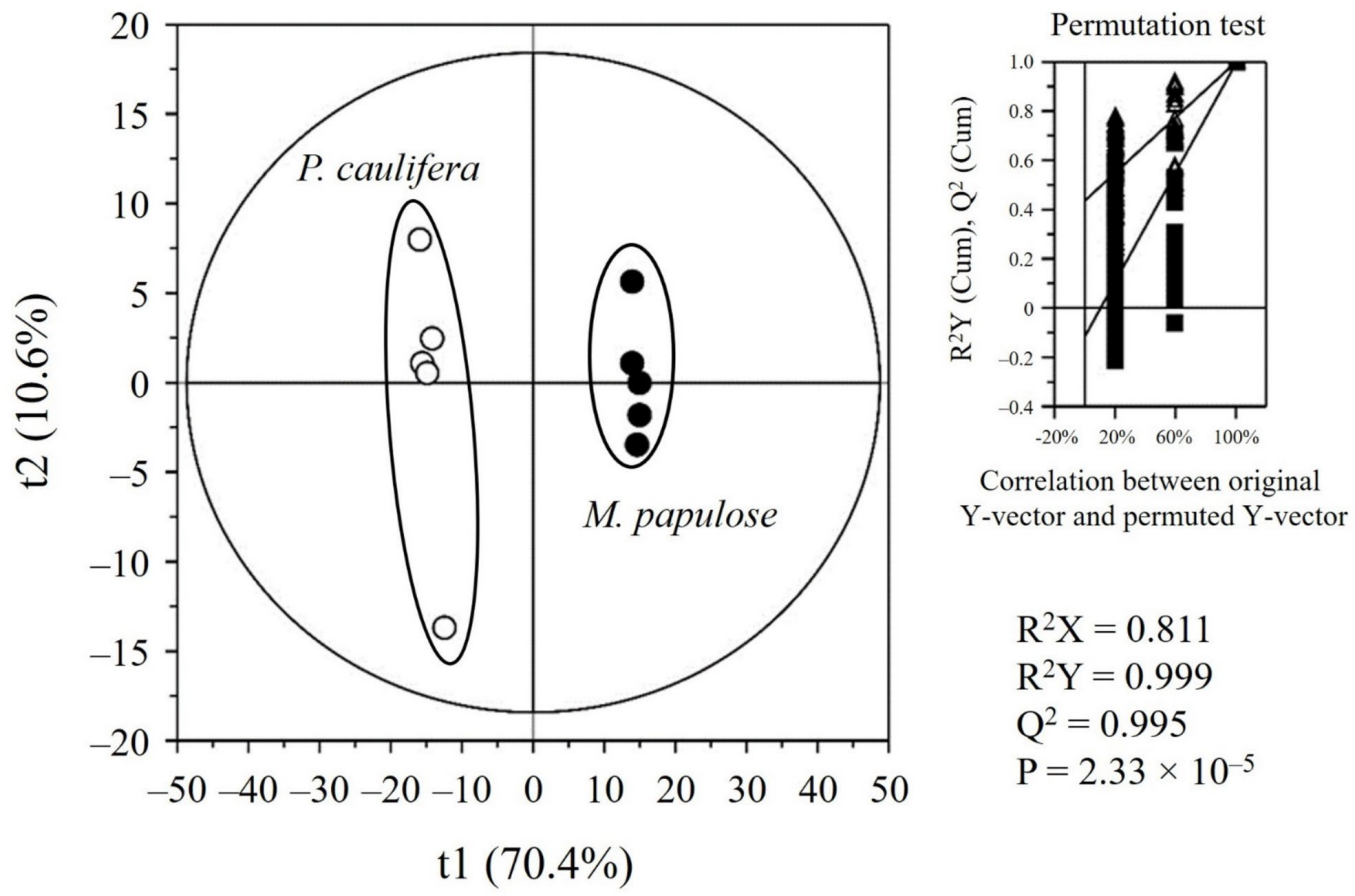
Seaweeds metabolites analysis. Partial least-squares discriminant analysis (PLS-DA) score plot of seaweed metabolites analyzed using ultra-performance liquid chromatography-quadrupole-time-of-flight mass spectrometry. The statistical acceptance of the PLS-DA model was evaluated using *R*^2^*X*, *R*^2^*Y*, *Q*^2^, and the *p-*value and validated using a permutation test and cross-validation (*n* = 200).

To investigate the differential metabolic effects of PCE and MPE, their metabolite profiles were analyzed using UPLC-QTOF MS. Distinctions between the samples were visualized using a partial least squares-discriminant analysis (PLS-DA) score plot (Figure 7). To identify the key metabolites contributing to this separation, the variable importance for projection (VIP) scores and *p*-values were calculated. Thirty metabolites were identified based on VIP values (>0.87) and p-values (<0.05). These included sulfur compounds (e.g., choline sulfate, 2-aminobutyl hydrogen sulfate, and glyceryl sulfoquinovoside), acidic compounds (e.g., 8-amino-7-oxononanoic acid, azelaic acid, and dimethyl azelate), amino acid derivatives (e.g., valyl isoleucine and caproyl sarcosine), lipids (e.g., C16 sphingamine, 4-hydroxy sphinganine(C17), lauryl betaine, oxohexadecanoic acid, and methyl hexadecanamide), aromatic compounds (e.g., monobutyl phthalate), phenolic compounds (e.g., dalbergin and ascosalopyrine), terpenes (e.g., chlorellatin A and dihydrobotrydial), prostaglandin E1, and pigments (e.g., 13b(s)-hydroxy-17c-ethoxypheophorbide a) (Supplementary Table S3 and Supplementary Figure 1). However, metabolites with m/z values of 223.0714, 482.3607, 715.3153, 282.2803, and 531.4075 (positive mode) and 124.9908, 233.9394, 369.0966, 262.0932, 383.2068, 385.2222, and 287.2225 (negative mode) were not identified. The relative abundances of key metabolites in the PCE and MPE were compared, revealing distinct fold changes (Supplementary Table S3).

### 3.9 Correlation analysis between metabolite profiles and physiological parameters

Spearman’s correlation analysis revealed significant associations between the metabolite profiles and key parameters in animal studies, cell studies, and gut microbiota composition. Several metabolites showed strong negative correlations with inflammatory markers (e.g., *Tnfα*, *Il-6*, and *Il-1β*) and adipocyte-related indicators (adipocyte size and BWG), including carnitine, choline sulfate, and valyl isoleucine, with high significance (*p* < 0.0001). Conversely, metabolites such as inosine, dalbergin, and certain unknown metabolites exhibited strong positive correlations with these parameters, which were also highly significant (p < 0.0001). Notably, metabolites that negatively correlated with inflammatory and adipocyte-related markers in animal studies exhibited positive correlations in cell studies, indicating opposite correlation patterns and discrepancies between *in vivo* and *in vitro* results. Gut microbiota-related parameters, particularly *Faecalibaculum* and *Dubosiella*, demonstrated robust correlations with multiple metabolites, suggesting a potential link between microbial composition and metabolite-driven physiological effects.

## 4 Discussion

**Figure 8:**
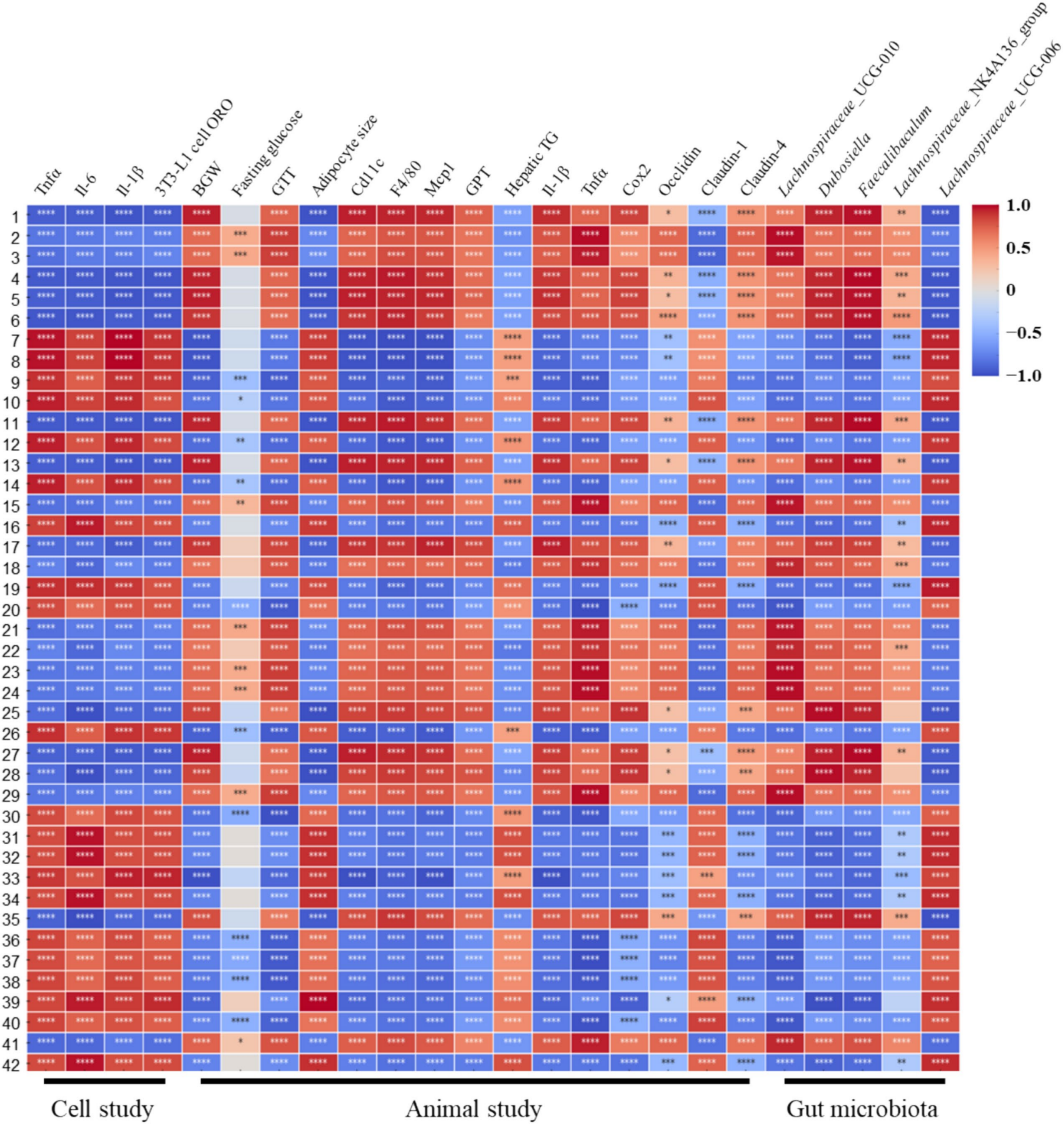
Spearman’s correlation heatmap showing associations between metabolite profiles (rows) and parameters from animal studies, cell studies, and gut microbiota (columns). Colors represent the strength and direction of correlations (red: positive, blue: negative). ^*^p < 0.05; ^**^p < 0.01; ^***^p < 0.001, ^****^p < 0.0001. 1, Choline sulfate; 2, 2-aminobutyl hydrogen sulfate; 3, glyceryl sulfoquinovoside; 4, carnitine; 5, ethosuximide M5; 6, valyl isoleucine; 7, inosine; 8, unknown 1; 9, unknown 2; 10, dalbergin; 11, unknown 3; 12, unknown 4; 13, ascosalipyrone; 14, unknown 5; 15, 8-amino-7-oxononanoic acid; 16, unknown 6; 17, 3-(3,5-dimethylphenoxy)-1,2-propanediol; 18, azelaic acid; 19, unknown 7; 20, chlorellatin A; 21, dimethyl azelate; 22, caproyl sarcosine; 23, dehydroabietic acid; 24, prostaglandin E1; 25, C16 sphingamine; 26, 4-hydroxyl sphinganine(C17); 27,dihydrobotrydial; 28, lauryl betaine; 29, monobuthy phthalate; 30, 2-amino-3-hexadecoxy-propan-1-ol;31,3,5-Bis[(3-methylbutanoyl)amino]-N-(2-methyl-2-propanyl) benzamide; 32, oxohexadecanoic acid; 33, unknown 8; 34, kribelloside C; 35, unknown 9; 36, lauryl pyrrolidone; 37, crucigasterin E; 38, 13b(S)-hydroxy-17c-ethoxypheaophorbide a; 39, unknown 10; unknown 11; 41, methyl hexadecanamide; 42, unknown 12.

Seaweed extracts or seaweed-derived polysaccharides can mitigate HFD-induced obesity and insulin resistance by altering the gut microbiota composition (6, 12, 14). However, the specific types of extracts that are most effective in reducing excessive fat expansion and suitable for use as supplements for obesity control remain unclear. To address this, we screened 10 different seaweed extracts (three green algae extracts, three brown algae extracts, and four red algae extracts), focusing on those cultivated in response to the climate crisis, in a lipid accumulation inhibition assay. PCE and MPE were the most potent extracts for reducing lipid accumulation during the different phases of adipocyte differentiation (Supplementary Figure 2A). Following this initial screening, we evaluated the effects of PCE and MPE on obesity using an HFD-induced obesity animal model supplemented with PCE or MPE for 8 weeks. HFD-induced obesity and its associated metabolic complications, including abnormal glucose metabolism (Figure 1), adipocyte hypertrophy and inflammation (Figure 2), metabolic-associated fatty liver disease (Figure 3), and gut barrier dysfunction (Figure 4), were significantly ameliorated by seaweed extract supplementation. Additionally, gut microbiota analysis revealed enhanced microbial diversity and beneficial shifts in microbial composition, including an increased abundance of *Lachnospiraceae* by PCE and MPE (Figure 5). Among the observed health benefits, improvements in hyperglycemia, adipocyte inflammation, hepatic lipid accumulation, inflammation, and tight junction integrity in the colon were more pronounced in the PCE-supplemented group than those in the MPE group. These robust effects of PCE were further corroborated by *in vitro* findings, where PCE exhibited stronger anti-inflammatory activity and greater reduction in lipid accumulation than those of MPE (Figure 6). Metabolomic analysis revealed distinct profiles for PCE and MPE, with PCE showing a higher relative abundance of specific metabolites (Figures 7,8; Supplementary Table S3; Supplementary Figure 1). These findings highlight the potential therapeutic applications of PCE and MPE in obesity (excessive fat accumulation), gut barrier dysfunction, and related metabolic complications. This study provides critical insights into the multifaceted roles of red seaweed extracts in promoting metabolic health, emphasizing their potential as effective interventions for HFD-induced metabolic disorders. We focused on three critical aspects: (1) the effects of PCE and MPE on HFD-induced inflammation, (2) improvement of gut permeability by PCE and MPE supplementation, and (3) the superior action of PCE compared with MPE in the context of metabolomic analysis.

The observed decrease in adipocyte size and CLS in visceral fat following supplementation with PCE and MPE suggests a promising anti-obesity effect (Figure 2). This finding is particularly relevant in the context of escalating global obesity rates and associated health challenges. The distinct responses of PCE and MPE to HFD-induced hyperglycemia and hyperinsulinemia provide insights into their metabolic effects (Figure 1). Although PCE significantly reduced both hyperglycemia and hyperinsulinemia, MPE primarily affected hyperinsulinemia, indicating potential differences in the mechanisms underlying their metabolic actions. Numerous bioactive extracts from marine algae reduce adiposity, inflammation, and abnormal glucose metabolism, often through mechanisms involving reduction in body weight, adiposity, and MAFLD, thereby improving insulin resistance (16, 17). PCE and MPE reduced inflammatory mRNA levels in adipose tissue and the liver (Figures 2, 3) without affecting liver mass, suggesting a targeted anti-inflammatory effect independent of adiposity and hepatic tissue weight reduction. This unique mechanism may play a critical role in the mitigation of obesity-associated metabolic complications. However, the specific bioactive components and precise inflammatory pathways inhibited by PCE and MPE remain unknown. Kim et al. demonstrated that *Sargassum horneri* ethanol extract containing sterols and gallic acid reduces particulate matter-induced oxidative stress in mouse lung cells via M1/M2 alveolar macrophage polarization. Astaxanthin, red-colored carotenoid pigments in algae, inhibits TGFβ1-induced Smad3 activation, consequently repressing the expression of fibrogenic genes. The impact of seaweed bioactive on inflammation is mostly through NF-kB related pathway (34–36). Thus, some bioactive components in PCE and MPE, especially PCE, may be the key regulators to reduce inflammation and overall obesity-mediated metabolic complications via modulation of NF-kB pathway. Given the metabolomic profiling performed (Figures 7, 8; Supplementary Table S3, and Supplementary Figure 1), further investigation is warranted to identify the active components of PCE that are most responsible for its beneficial actions and elucidate the mechanisms driving these effects.

The restoration of tight junction protein expression, specifically *occludin* and *claudin-1*, underscores the protective role of these seaweed extracts in preserving intestinal barrier integrity (Figure 4). The prevention of gut barrier disruption in HFD-fed mice suggests a potential mechanism by which PCE and MPE influence systemic metabolic health. Moreover, the effects of PCE and MPE on the gut microbiota (Figure 5) introduce an additional layer of complexity to their potential health benefits. The increase in the abundance of the native protective bacterium, *Lachnospiraceae*, recognized for its butyrate-producing capabilities and protective effects against colorectal cancer, aligns with the increasing understanding of the role of gut microbiota in metabolic health (33). The modulation of the gut microbiota by PCE and MPE suggests a holistic approach to address obesity and its associated complications. In our study, we also conducted anti-adipogenic screening of various components in red seaweed, such as *κ*-carrageenan (K-CA) and *ι*-carrageenan (I-CA) polysaccharide and R-phycoerythrin (R-PE), a bright red phycobiliprotein found in red algae. However, no significant differences were observed in the lipid-lowering effects during adipogenesis (Supplementary Figure 2B). Seaweed-derived polysaccharides modulate the gut microbiota, which consequently improves metabolic complications, mostly due to the beneficial effects of fiber and polysaccharides, such as inulin (6, 14). These findings may explain the reason PCE exhibit more robust beneficial actions *in vitro* compared with those of MPE. Specific components of PCE are primarily responsible for its anti-obesity effects and synergistic interactions among its bioactive compounds enhance its efficacy. Additionally, the enhanced abundance of *Lachnospiraceae*, butyrate-producing bacteria, is strongly correlated with beneficial health outcomes, such as improved gut integrity and meta-inflammation. To address these hypotheses, future studies involving bioavailability assays and efficacy tests for both PCE and MPE are warranted to elucidate their mechanisms of action.

Metabolite profiling identified distinct metabolic signatures for PCE and MPE, as depicted in the PLS-DA score plot. These findings highlight the unique metabolomic characteristics of PCE and MPE, with specific metabolites contributing to the distinctive properties of each extract. Although PCE and MPE are derived from edible seaweeds, supplements based on these extracts are often consumed at levels typically approved in Australia for beneficial uses, akin to drug-like applications. Typical recommendations for extracts, such as fucoidan or polysaccharides (37) range from 300 to 1,000 mg/day for general health effects in a 70 kg adult male, equivalent to approximately 4.28–14.28 mg/kg BW. Based on these calculations, the 10 mg/kg BW doses of PCE and MPE used in this study were within a comparable range, despite being whole extracts rather than isolated polysaccharides. However, the 10 mg/kg BW dose used in animal experiments, while relatively modest for such studies, presents challenges for translation to human physiology, owing to differences in metabolism and bioavailability. The bioavailability of PCE and MPE have not yet been reported, which is a primary limitation of this study. This gap in evidence restricts the rationale for selecting 10 mg/kg BW for oral administration in animals and 10 μg/mL for *in vitro* experiments. Although the extracts were prepared via ethanol extraction from thoroughly washed seaweed biomass-a process expected to substantially reduce inorganic salt content-residual sodium and iodine, were not directly quantified. Although dietary salt is well known to influence cardiovascular health, its direct effects on adiposity, insulin sensitivity, and energy homeostasis remain poor. Accordingly, residual salt is unlikely to be the primary driver of the metabolic improvements observed here, which are more plausibly attributable to the bioactive constituents of PCE and MPC. Additionally, given the iodine content of typical seaweed extract doses, toxicology studies in animals and short-term clinical trials are essential to ensure its safety and efficacy. The divergent correlation patterns between metabolites observed in animal and cell studies suggest important physiological and mechanistic differences between *in vivo* and *in vitro* environments (38). These discrepancies could be attributed to variations in metabolism, absorption, and bioavailability, which are intrinsic to living organisms but absent in isolated cell systems (39). Given that the bioavailability of PCE and MPE remains uncharacterized, translating the 10 mg/kg BW dosage used in animal experiments to clinically relevant human doses pose substantial challenges (40, 41) and The 10 mg/kg PCE and MPE dose was empirically selected with reference to human-relevant intake ranges in the absence of quantified active constituents and *in vivo* exposure data, and our study lacks a formal dose–response evaluation. Future work will incorporate constituent identification and LC–MS/MS–based standardization, alongside pharmacokinetic and pharmacodynamic studies to refine dosing and strengthen translational relevance. Additionally, metabolites that show distinct correlation patterns may act differently in the complex metabolic environments of living organisms compared with control cell cultures (42, 43). Despite these differences in the correlation patterns between the cell and animal studies, the results clearly demonstrated the efficacy of both extracts, reinforcing their therapeutic potential against obesity and related metabolic disorders.

Despite the limitations, to the best of our knowledge, this study is the first to demonstrate the efficacy of two distinct red algae extracts, predominantly found in Jeju, in improving obesity-related outcomes. Our study elucidated the multifaceted effects of PCE and MPE on the inhibition of diet-induced obesity and the alteration of metabolism, intestinal barrier function, and gut microbiota composition. The distinctive responses of these extracts to metabolic parameters underscore the importance of considering specific seaweed species when developing targeted interventions. Further exploration of the bioavailability and dose–response, and duration-response effects, underlying molecular mechanisms, and long-term effects of PCE and MPE is essential to fully understand their therapeutic potential and potential applications in combating obesity-related metabolic disorders.

## 5 Conclusion

This study demonstrates that PCE and MPE supplementation effectively alleviates HFD-induced metabolic dysfunction through coordinated regulation of adipose tissue, liver, intestinal barrier integrity, and gut microbiota composition. Both extracts reduced adipocyte hypertrophy and inflammation, attenuated hepatic steatosis and liver injury, and improved systemic metabolic parameters. These benefits were accompanied by modulation of tight junction protein expression and shifts in gut microbial taxa associated with improved metabolic and inflammatory profiles. Despite these shared effects, PCE and MPE exhibited distinct biological activities. PCE showed greater efficacy in lowering fasting glucose levels, reducing hepatic triglyceride accumulation, and restoring intestinal tight junction protein expression in vivo. Consistently, PCE exerted stronger anti-inflammatory and anti-adipogenic effects *in vitro*, more effectively suppressing LPS-induced cytokine expression in macrophages and lipid accumulation during adipocyte differentiation than MPE. Metabolomic analyses revealed distinct chemical signatures between PCE and MPE, with differential enrichment of bioactive metabolites linked to inflammation, metabolism, and gut microbial interactions. Collectively, these findings highlight PCE as a particularly promising functional ingredient and advance the understanding of seaweed-derived bioactive in the management of obesity-associated metabolic disorders.

## Data Availability

The 16S rRNA sequencing data are available in the NCBI Sequence Read Archive (SRA) under BioProject accession number PRJNA1439618.

## Footnotes

1
http://metlin.scripps.edu

2
www.hmdb.ca
