# Supplementary material for: Marine-derived extracts of Peyssonnelia caulifera Okamura and Meristotheca papulosa demonstrate differential efficacy in modulating obesity-related metabolic skewing revealed by integrative analysis of extract metabolomics and microbiome profiles
Source: Front Nutr. 2026 Apr 9;13:1749413. doi: 10.3389/fnut.2026.1749413 (PMC13104251; doi:10.3389/fnut.2026.1749413)
Supplement: Supplementary file 1 [file Supplementary_file_1.docx]

Supplementary Material

# Supplementary Tables

**Table S1. Dietary composition**

| **Ingredients** | **LF** | **HF** |
| --- | --- | --- |
|  | g/kg | g/kg |
| Casein | 200 | 200 |
| l-Cysteine | 3 | 3 |
| Sucrose | 100 | 69 |
| Corn starch | 397 | 0 |
| Maltodextrin 10 | 132 | 125 |
| Lard | 0 | 245 |
| Soybean oil | 70 | 25 |
| Cellulose | 50 | 50 |
| Mineral mix | 35 | 35 |
| Calcium phosphate | 3.4 | 3.4 |
| Vitamin mix | 10 | 10 |
| Choline bitartrate | 2.5 | 2 |
| Total | 1003 | 784 |
|  | (3.9 kcal/g) | (5.2 kcal/g) |
|  |  |  |
|  | kcal (%) | kcal (%) |
| Carbohydrate | 64 | 20 |
| Protein | 20 | 19 |
| Fat | 16 | 61 |
|  | 100 | 100 |

The AIN-93G diet was modified. HF, high-fat; LF, low-fat

**Table S2. Primer sequences**

| **Gene** | **5ʹLeft (Forward)3ʹ** | **5ʹLeft (Forward)3ʹ** |
| --- | --- | --- |
| m*Cd11c* | CTGGATAGCCTTTCTTCTGCTG | GCACACTGTGTCCGAACTC |
| m*F4/80* | CTTTGGCTATGGGCTTCCAGTC | GCAAGGAGGACAGAGTTTATCGTG |
| m*Mcp1* | AGGTCCCTGTCATGCTTCTG | GCTGCTGGTGATCCTCTTGT |
| m*Cox2* | AAAGGTTCTTCTACGGAGAGAGTTCA | TGGGCAAAGAATGCAAACATC |
| m*Tnfα* | GGCTGCCCCGACTACGT | ACTTTCTCCTGGTATGAGATAGCAAAT |
| m*Il-1ß* | AAATACCTGTGGCCTTGGGC | CTTGGGATCCACACTCTCCAG |
| m*Occludin* | GCTATGGAGGCTATGGCTATGG | CTAAGGAAGCGATGAAGCAGAAG |
| m*Claudin-4* | TGGGGACAGGCAAACCCGGA | CTTGCCGGCCGTAAGGAGCC |
| m*Zo-1* | AGGACACCAAAGCATGTGAG | GGCATTCCTGCTGGTTACA |
| m*Rplp0* (*36b4*) | GGATCTGCTGCATCTGCTTG | GGCGACCTGGAAGTCCAACT |
| m*Hprt* | TTGCTCGAGATGTCATGAAGGA | AGCAGGTCAGCAAAGAACTTATAGC |

**Table S3.** Identification of major metabolites contributing to differences between sample groups using UPLC-Q-TOF MS.

| No. | RT | Compounds | Adduct | Exact mass  (m/z) | Mass fragment | VIP | P-value | Fold change (*vs. M. papulose*) |
| --- | --- | --- | --- | --- | --- | --- | --- | --- |
| 1 | 0.68 | choline sulfate | M+H | 184.0642 | 104 | 1.17 | 5.01 × 10^−7^ | 8907.0 |
| 2 | 0.71 | 2-aminobutyl hydrogen sulfate | M-H | 168.033 | 96, 79, 124, 152 | 1.18 | 8.18 × 10^−8^ | 470.8 |
| 3 | 0.74 | glyceryl sulfoquinovoside | M-H | 317.054 | 80, 225, 94, 79, 164 | 1.10 | 1.25 × 10^−4^ | 9.8 |
| 4 | 0.74 | carnitine | M+H-H_2_O | 144.1007 | 116, 100 | 1.19 | 8.36 × 10^−12^ | 30.9 |
| 5 | 0.74 | ethosuximide M5 | M+H-H_2_O | 138.0541 | 121, 83, 81 | 1.18 | 6.53 × 10^−8^ | 13.9 |
| 6 | 0.75 | valyl isoleucine | M+H | 231.1707 | 144, 84, 130, 155 | 1.19 | 6.23 × 10^−11^ | 3206.6 |
| 7 | 0.76 | inosine | M-H | 267.072 | 135, 133 | 1.15 | 8.83 × 10^−6^ | −37.8 |
| 8 | 0.76 | unknown 1 | M-H | 124.9908 |  | 1.19 | 1.90 × 10^−12^ | ND |
| 9 | 2.30 | unknown 2 | M-H | 233.9394 |  | 1.19 | 1.30 × 10^−11^ | ND |
| 10 | 2.31 | dalbergin | M-H | 267.0731 | 252, 224, 180 | 0.88 | 1.25 × 10^−2^ | 13882.5 |
| 11 | 2.44 | unknown 3 | M+H | 223.0714 |  | 1.19 | 2.11 × 10^−10^ | ND |
| 12 | 3.00 | unknown 4 | M-H | 369.0966 |  | 1.17 | 1.46 × 10^−7^ | + |
| 13 | 3.06 | ascosalipyrone | M+H | 239.1292 | 221, 197, 175 | 1.18 | 1.15 × 10^−8^ | −7758.8 |
| 14 | 3.23 | unknown 5 | M-H | 262.0932 |  | 1.19 | 2.00 × 10^−13^ | 1.2 |
| 15 | 3.51 | 8-amino-7-oxononanoic acid | M-H | 186.1127 | 125, 168, 123 | 1.04 | 4.46 × 10^−4^ | ND |
| 16 | 3.98 | unknown 6 | M-H | 383.2068 |  | 1.19 | 5.09 × 10^−12^ | 9.5 |
| 17 | 3.99 | 3-(3,5-dimethylphenoxy)-1,2-propanediol | M+H | 197.1172 | 179, 161, 107 | 1.16 | 1.20 × 10^−6^ | 2.8 |
| 18 | 4.07 | azelaic acid | M-H | 187.0967 | 125, 97, 171 | 0.87 | 1.50 × 10^−2^ | ND |
| 19 | 4.23 | unknown 7 | M-H | 385.2222 |  | 1.19 | 4.83 × 10^−9^ | −1.6 |
| 20 | 4.80 | chlorellatin A | M+H | 219.1747 | 2023, 163, 133 | 1.18 | 8.84 × 10^−8^ | 26.3 |
| 21 | 4.82 | dimethyl azelate | M-H | 215.129 | 185, 152 | 1.17 | 5.32 × 10^−7^ | 2.6 |
| 22 | 4.92 | caproyl sarcosine | M-H | 242.176 | 224 | 1.01 | 1.89 × 10^−3^ | + |
| 23 | 5.02 | dehydroabietic acid | M+H | 301.213 | 131, 145 | 1.18 | 2.35 × 10^−7^ | 2217.6 |
| 24 | 5.06 | prostaglandin E1 | M-H | 353.233 | 309, 193, 291, 171, 263, 211, 181 | 1.18 | 3.84 × 10^−8^ | 1.3 |
| 25 | 5.28 | C16 sphingamine | M+H | 274.2746 | 217, 159 | 1.14 | 1.12 × 10^−5^ | ND |
| 26 | 5.51 | 4-hydroxy sphinganine(C17) | M+H | 304.2847 | 256, 286 | 1.19 | 6.53 × 10^−12^ | + |
| 27 | 5.64 | dihydrobotrydial | M-H | 311.180 | 267, 293 | 0.91 | 9.19 × 10^−3^ | 1.3 |
| 28 | 5.74 | lauryl betaine | M+H | 272.2573 | 104 | 1.11 | 6.58 × 10^−5^ | 3847.3 |
| 29 | 5.87 | monobutyl phthalate | M-H | 221.080 | 177, 121, 120 | 1.03 | 1.29 × 10^−3^ | −1.2 |
| 30 | 5.98 | 2-amino-3-hexadecoxy-propan-1-ol | M+H | 316.3212 | 272, 106 | 1.11 | 9.21 × 10^−5^ | −7.1 |
| 31 | 6.00 | 3,5-Bis[(3-methylbutanoyl)amino]-N-(2-methyl-2-propanyl)benzamide | M-H | 374.243 | 232, 316, 189 | 1.04 | 4.46 × 10^−4^ | ND |
| 32 | 6.62 | oxohexadecanoic acid | M-H | 269.210 | 125, 199, 111 | 1.06 | 6.04 × 10^−4^ | −212.0 |
| 33 | 6.93 | unknown 8 | M-H | 287.2225 |  | 1.19 | 1.57 × 10^−17^ | −19.1 |
| 34 | 7.20 | kribelloside C | M+HCOO^-^ | 699.383 | 255, 397, 241 | 0.98 | 2.08 × 10^−3^ | 37.1 |
| 35 | 7.45 | unknown 9 | M+H | 482.3607 |  | 1.19 | 1.12 × 10^−10^ | −2.6 |
| 36 | 7.79 | lauryl pyrrolidone | M+H | 254.2466 | 212 | 0.95 | 6.46 × 10^−3^ | −3.6 |
| 37 | 7.99 | crucigasterin E | M+H | 280.2618 | 245, 147, 105, 133, 161 | 1.00 | 2.50 × 10^−3^ | −13.2 |
| 38 | 8.27 | 13b(S)-hydroxy-17c-ethoxypheaophorbide a | M+H | 637.3015 | 619, 577 | 1.14 | 1.77 × 10^−5^ | −15.2 |
| 39 | 8.27 | unknown 10 | M+H | 715.3153 |  | 1.19 | 4.32 × 10^−11^ | −4.0 |
| 40 | 8.47 | unknown 11 | M+H | 282.2803 |  | 1.17 | 2.32 × 10^−7^ | + |
| 41 | 8.69 | methyl hexadecanamide | M+H | 270.2787 | 154, 88, 102 | 1.14 | 3.12 × 10^−5^ | ND |
| 42 | 8.77 | unknown 12 | M+H | 531.4075 |  | 1.16 | 1.77 × 10^−6^ | + |

P-values were analyzed using Duncan’s test. RT, retention time; VIP, variable importance for projection; ND, not detected.

**Table S4.** Characteristic of diet intake, metabolic parameters and blood lipid profiles*

| **Group** | **LF** | **HF** | **HF+PCE** | **HF+MPE** | **P-value** |
| --- | --- | --- | --- | --- | --- |
| **Diet intake** |  |  |  |  |  |
| Food intake (g/mouse/day) | 4.65±0.26 ^a^ | 2.38±0.12 ^b^ | 2.23±0.13 ^b^ | 2.32±0.16 ^b^ | 0.0001 |
| Kcal intake (kcal/mouse/day) | 18.54±1.04 ^a^ | 12.59±0.63 ^b^ | 11.79±0.69 ^c^ | 12.27±0.85 ^c^ | 0.0001 |
| FER (%) | 7.81±0.57 ^c^ | 32.69±3.84 ^a^ | 26.94±3.07 ^ab^ | 19.09±2.97 ^b^ | 0.0001 |
| **Phenotypes** |  |  |  |  |  |
| Initial BW (g) Final BW (g) | 23.44±0.29 ^b^ 29.82±0.47 ^c^ | 25.13±0.57 ^a^ 38.31±1.67 ^a^ | 24.47±0.31 ^ab^ 34.33±1.22 ^b^ | 24.61±0.42 ^ab^ 34.03±0.95 ^b^ | P<0.05 0.0001 |
| BW gain (g) | 6.38±0.44 ^b^ | 11.57±2.00 ^a^ | 9.86±1.15 ^ab^ | 9.41±0.85 ^ab^ | 0.001 |
| Liver (g) | 0.98±0.03 | 1.09±0.07 | 0.95±0.04 | 0.98±0.06 | *n.s*. |
| Liver/BW (%) | 100.00±2.64 ^a^ | 85.33±3.37 ^b^ | 83.39±1.30 ^b^ | 86.78±3.71 ^b^ | 0.0001 |
| Epididymal fat (g) | 1.03±0.13 ^b^ | 2.11±0.20 ^a^ | 1.76±0.18 ^a^ | 1.90±0.21 ^a^ | 0.001 |
| Epididymal fat/BW (%) | 100±11.52 ^b^ | 161.02±12.41^a^ | 148.92±13.38 ^a^ | 162.27±15.14 ^a^ | 0.005 |
| **Blood Chemistry** |  |  |  |  |  |
| Triglyceride (mg/dL) | 61.11±4.79 | 59.13±3.11 | 48.20±5.67 | 54.88±5.13 | *n.s*. |
| Total Cholesterol (mg/dL) | 110.91±6.76 ^b^ | 144.18±13.91 ^a^ | 106.3±7.54 ^b^ | 139.80±10.80 ^a^ | P<0.05 |

* Values are presented as mean ± SEM (n = 8–10 per group). Statistical differences were analyzed by one-way ANOVA followed by Tukey’s post hoc test. Different superscript letters (a, b, c) indicate significant differences between groups (p < 0.05); values sharing the same letter are not significantly different. FER, food efficiency ratio (body weight gain / food intake × 100). Liver/BW (%) and epididymal fat/BW (%) values were normalized to the LF group average and set to 100%. n.s., not significant. LF, low-fat diet; HF, high-fat diet; PCE, *Peyssonnelia caulifera Okamurae* extract; MPE, *Meristotheca papulosa* extract; BW, body weight.

# Supplementary Figures


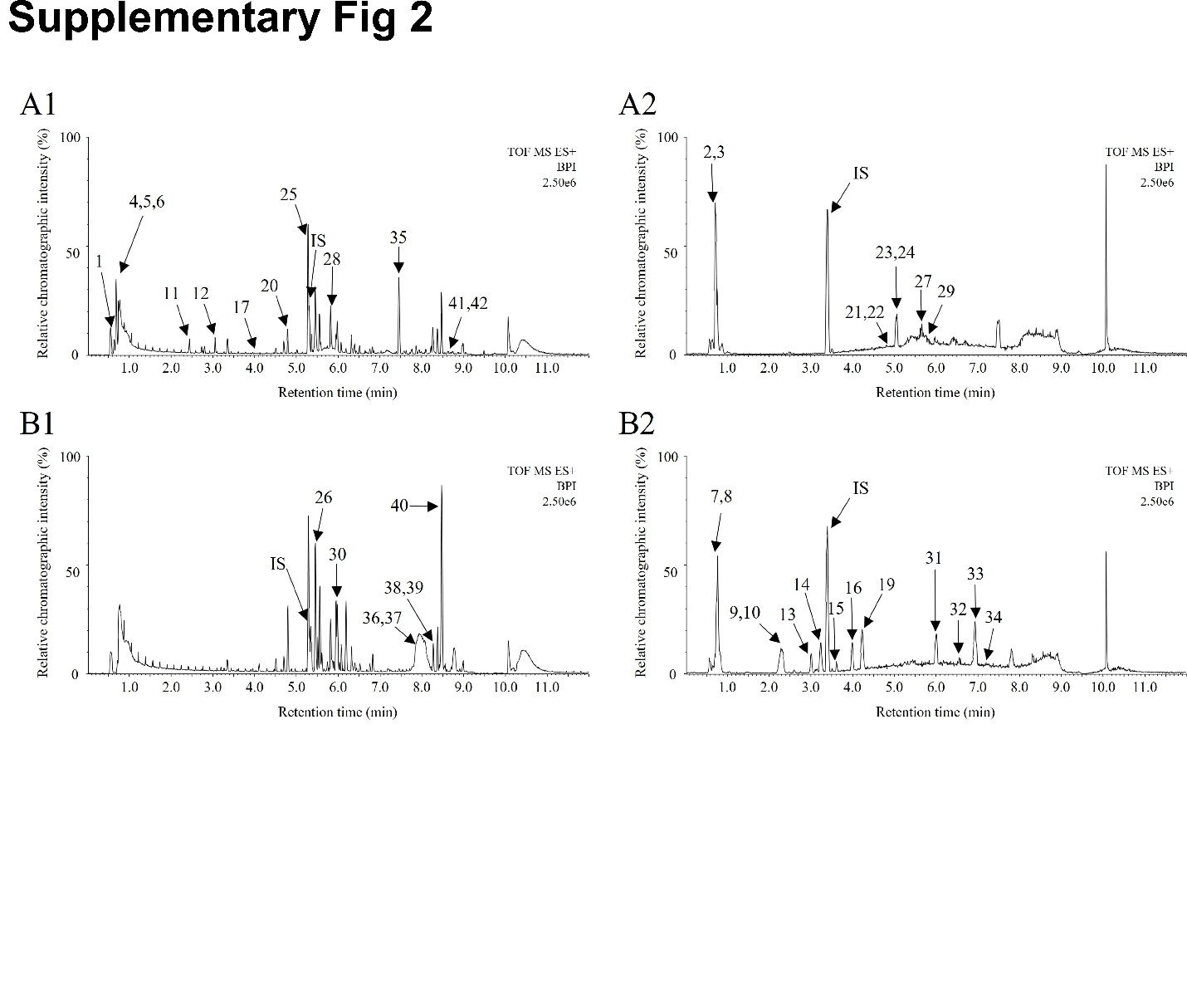


**Supplementary Figure 1.** Representative chromatograms of global metabolites in *P. caulifera* (A) and *M. papulose* (B) analyzed by ultra-performance liquid chromatography-quadrupole-time-of-flight mass spectrometry (A1 and B1: positive mode and A2 and B2: negative mode). 1, choline sulfate; 2, 2-aminobutyl hydrogen sulfate; 3, glyceryl sulfoquinovoside; 4, carnitine; 5, ethosucimide M5; 6, valyl isoleucine; 7, inosine; 8, unknown 1; 9, unknown 2; 10, dalbergin; 11, unknown 3; 12, ascosalipyrone; 13, unknown 4; 14, unknown 5; 15, 8-amino-7-oxononanoic acid; 16, unknown 6; 17, 3-(3,5-dimethylphenoxy)-1,2-propanediol; 18, azelaic acid; 19, unknown 7; 20, chlorellatin A; 21, dimethyl azelate; 22, caproyl sarcosine; 23, dehydroabietic acid; 24, prostaglandin E1; 25, C16 sphingamine; 26, 4-hydroxyl sphinganine(C17); 27, dihydrobotrydial; 28, lauryl betaine; 29, monobutyl phthalate; 30, 2-amino-3-hexadecoxy-propan-1-ol, 31, 3,5-Bis[(3-methylbutanoyl)amino]-N-(2-methyl-2-propanyl)benzamide; 32, oxohexadecanoic acid; 33, unknown 8; 34, kribelloside C; 35, unknown 9; 36, lauryl pyrrolidone; 37, crucigasterin E; 38, 13b(S)-hydroxy-17c-ethoxypheaophorbide a; 39, unknown 10; 40, unknown 11; 41, methyl hexadecanamide; 42, unknown 12; IS, internal standard.


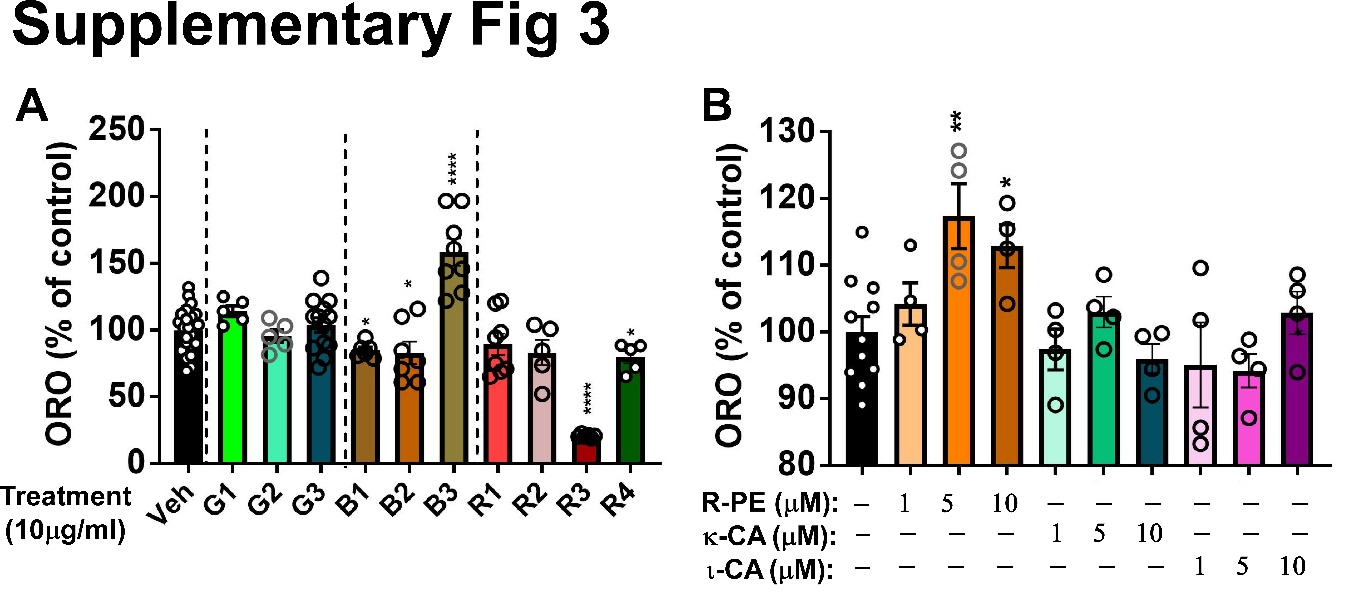


**Supplementary Figure 2.** The 3T3-L1 cells were seeded and induced to differentiation in the presence of DMSO (vehicle control), G (Green algae extracts) 1, G2, G3, B (Brown algae extracts) 1, B2, B3, R (Red algae extracts) 1, R2, R3, or R4 (10 μg/mL) for 7 days. (A) TG accumulation was visualized by Oil red O staining and its intensity was measured by microplate reader (500 nm), 3T3-L1 cells were seeded and induced to differentiation in the presence of DMSO (vehicle control), R-PE, κ-CA, or ι-CA (1–10 μM) for 7 days. (B) TG accumulation was visualized by Oil red O staining and its intensity was measured by microplate reader (500 nm). Data are expressed as the mean ± SEM. * P<0.05, ** P<0.01, **** P<0.0001 (vehicle vs. treatment) by one-way ANOVA with Bonferroni's comparison test or student t-test. ORO, Oil red O stain; TC, total cholesterol; TG, triglyceride
